# ncRNAs: New Players in Mitochondrial Health and Disease?

**DOI:** 10.3389/fgene.2020.00095

**Published:** 2020-02-28

**Authors:** Mirjana Gusic, Holger Prokisch

**Affiliations:** ^1^ Institute of Human Genetics, Helmholtz Zentrum München, Neuherberg, Germany; ^2^ DZHK (German Centre for Cardiovascular Research), partner site Munich Heart Alliance, Munich, Germany; ^3^ Institute of Human Genetics, Technical University of Munich, Munich, Germany

**Keywords:** mitochondria, ncRNA, lncRNA, miRNA, mtDNA, micropeptide

## Abstract

The regulation of mitochondrial proteome is unique in that its components have origins in both mitochondria and nucleus. With the development of OMICS technologies, emerging evidence indicates an interaction between mitochondria and nucleus based not only on the proteins but also on the non-coding RNAs (ncRNAs). It is now accepted that large parts of the non‐coding genome are transcribed into various ncRNA species. Although their characterization has been a hot topic in recent years, the function of the majority remains unknown. Recently, ncRNA species microRNA (miRNA) and long-non coding RNAs (lncRNA) have been gaining attention as direct or indirect modulators of the mitochondrial proteome homeostasis. These ncRNA can impact mitochondria indirectly by affecting transcripts encoding for mitochondrial proteins in the cytoplasm. Furthermore, reports of mitochondria-localized miRNAs, termed mitomiRs, and lncRNAs directly regulating mitochondrial gene expression suggest the import of RNA to mitochondria, but also transcription from the mitochondrial genome. Interestingly, ncRNAs have been also shown to hide small open reading frames (sORFs) encoding for small functional peptides termed micropeptides, with several examples reported with a role in mitochondria. In this review, we provide a literature overview on ncRNAs and micropeptides found to be associated with mitochondrial biology in the context of both health and disease. Although reported, small study overlap and rare replications by other groups make the presence, transport, and role of ncRNA in mitochondria an attractive, but still challenging subject. Finally, we touch the topic of their potential as prognosis markers and therapeutic targets.

## Background

Molecular biology has historically described RNA as an intermediate between genetic information stored in DNA and protein synthesis. The estimated number of protein-coding genes is around 20,000 (Pertea et al., 2018). Classical approaches to classify RNAs with protein-coding potential—the messenger RNAs (*mRNAs*)—were typically based on the existence of open reading frame (ORF) longer than 300 nucleotides (nt), conservation, and/or functional domains (Dinger et al., 2008). Nevertheless, as protein-coding regions encompass only ∼2% of the human genome, the rest has been considered as “dark matter”. Detected RNAs not translated into proteins were named non-coding RNAs (*ncRNA*) and initially regarded as a transcriptional noise or the byproducts of genetic information flow from DNA to protein. Nevertheless, since the discovery of transfer RNAs (tRNAs) and ribosomal RNAs (rRNAs), the number and understanding of new and putative functional ncRNAs have expanded. Moreover, the boundaries between the coding and non-coding RNAs have become more blurry. Evidence is emerging that some RNAs, initially classified as non-coding, hide small ORFs (sORFs, < 300 nt) encoding for small functional peptides- micropeptides. Currently, we know dozen of different ncRNAs, which can be can be classified as housekeeping or regulatory ncRNS, according to Szymanski et al. (2003).

*Housekeeping ncRNAs* are constitutively expressed and mostly well functionally characterized classes of rRNAs, tRNAs, small nuclear RNAs (snRNAs), small-nucleolar RNAs (snoRNAs), Ribonuclease P RNA (*RNase P*), Ribonuclease MRP RNA (*MRP RNase*, *RNRP*), and Telomerase RNA component (*TERC*). *rRNAs* are the most abundant class of RNAs in most cells, composing around 80% of cellular transcriptome. They serve as the essential binding site for ribosomal proteins within the assembled ribosome and contribute to the binding of extra-ribosomal factors and ribosome-associated proteins, resulting in the protein translation machinery (Noller et al., 2017; Simsek et al., 2017). *tRNAs* provide the interface between nucleic acids and proteins during translation by carrying an amino acid on its 3′ end and reading the mRNA by base-pairing induced by the ribosome, which uniquely determines the position of amino acids in proteins (Schimmel, 2018). *snRNAs* participate in the assembly and function of canonical spliceosomes (Wang and Burge, 2008). *snoRNAs* are localized to the nucleolus and guide the methylation and pseudouridylation of rRNAs, tRNAs, and snRNAs (Maxwell and Fournier, 1995). *RNase P* has a role in precursor-tRNA cleavage, *RMRP* in precursor-rRNA cleavage, and *TERC* in telomere synthesis (discussed later).

*Regulatory ncRNAs* are mostly produced in a cell- or tissue-specific fashion during certain stages of cell differentiation or organism development, or as a response to changes in the environment. They are still poorly understood and a very heterogeneous group that can act in different ways, from gene expression regulation to modulation of protein and RNA distribution within cells (Szymanski et al., 2003). They are divided based on their length into short (<200 nt) and long (>200 nt, lncRNAs) RNAs. *Short ncRNAs* consist of microRNAs (miRNAs), small interfering RNAs (siRNAs) and Piwi-associated RNAs (piRNAs). *miRNAs* are endogenous, single-stranded, 19-23 nt in length RNAs that can bind to a target mRNA with a complementary sequence to induce its cleavage, degradation, or interfere with translation. Similar in size, *siRNAs* are exogenous RNAs that undergo processing and function in post-transcriptional gene silencing (Carthew and Sontheimer, 2009). *piRNAs* are single stranded, 26-31 nucleotides long RNAs that form complexes with the piwi family of proteins. These complexes have a role in RNA and epigenetic silencing of transposons (Siomi et al., 2011). Longer than 200 nt, *lncRNAs* represent the most abundant, yet least understood class of RNAs, with an average length ~ 1000 nt (Ulitsky and Bartel, 2013). They share some features typical for mRNAs, such as transcription by the RNA-polymerase II (Pol II), 5′end cap, 3′end polyadenylation and presence of alternative splicing isoforms (Kopp and Mendell, 2018). However, compared to the mRNAs, they exhibit lower expression levels, more tissue-specific expression, and poor sequence conservation (Derrien et al., 2012; Djebali et al., 2012; Kopp and Mendell, 2018; Fazal et al., 2019). Although often considered as nucleus-enriched, lncRNAs exhibit variety of subcellular localization, which often helps to determine their biological function (Carlevaro-Fita and Johnson, 2019). Finally, *circular RNAs (circRNAs)* are a special class of RNAs with the 3′ and 5′ ends covalently linked, generally formed by alternative splicing of pre-mRNA (Salzman et al., 2012). They have been proposed to act as miRNAs sponges or even as templates for protein synthesis (Ragan et al., 2019).

Interest in the ncRNAs has been stimulated by the development of high-throughput OMICS technologies. Genome‐, transcriptome‐, translatome- and proteome‐wide measurements by the whole genome sequencing (WGS), RNA-sequencing (RNA-seq), ribosome profiling (Ribo-seq) and mass spectrometry (MS), respectively. In combination, these methods offer the possibility of a systematic analysis of different stages of gene expression (Ori et al., 2015; Wang et al., 2019). RNA-seq data have shown that up to 85% of the genome is transcribed and identified, among others, novel transcript isoforms, transcripts arising from intergenic regions, overlapping transcripts, and transcribed pseudogenes (Consortium, 2012; Djebali et al., 2012; Hangauer et al., 2013). Ribo-seq has shown widespread and pervasive translation on cytosolic RNAs, with surprisingly ~40% lncRNAs being engaged with the ribosome (Ingolia et al., 2009; Kearse and Wilusz, 2017). Reported ribosomal occupancy of RNAs indicated on the one side presence of different protein isoforms and regulatory upstream open reading frames ORFs (uORFs) from the mRNAs, and on the other, more exciting side, new ways of translational regulation and possible micropeptide production from lncRNAs (Morris and Geballe, 2000; Andrews and Rothnagel, 2014). It must be taken into account that the ribosomal occupancy of transcripts need not automatically lead to the production of stable, functional polypeptides, and that further evidence is needed in order to reclassify transcripts as indeed protein-coding (Guttman et al., 2013). MS has proven as a useful tool to inspect the postulated translational event, with developing proteogenomics approaches confirming the presence of some peptides encoded by previously non-coding regions (Slavoff et al., 2013; Fields et al., 2015; Wang et al., 2019). However, in order to omit the possibility of false-positive findings from MS, further functional studies on revealed peptides are needed, and these studies remain sparse.

The complexity of gene expression has in most cases been published on the levels of detection and its functional relevance remains elusive. Still, it has revealed that the distinguishment between mRNAs and ncRNAs is more challenging than initially assumed and that automatic gene annotation systems, although straightforward across large datasets, can sometimes be misleading. Traditional arbitrary ORF cutoff can lead to misclassification of some ncRNAs as mRNAs as they can by chance contain putative ORFs. This is especially true for lncRNAs, such as functionally characterized *H19*, *Xist*, *Mirg*, *Gtl2*, and *KcnqOT1* (Prasanth and Spector, 2007). Some ncRNAs have evolved from the protein-coding genes, and so will keep certain features and homologies to mRNAs (Duret et al., 2006). For example, *Xist* has evolved into the ncRNA through the process of pseudogenization, during which proto-*Xist* had lost its protein-coding function and its flanking genes had turned into pseudogenes (Duret et al., 2006). On the contrary, micropeptide-encoding regions may be incorrectly classified as non-coding due to their size (Yeasmin et al., 2018). Next, the absence of ORF conservation does not guarantee an absence of protein-coding potential. Indeed, the majority of micropeptide-encoding regions are not conserved (Ji et al., 2015), suggesting their role in encoding evolutionary young proteins (Ruiz-Orera et al., 2014). Finally, some genes are bifunctional, and its products function independently both as RNAs and proteins. The first report of such a gene was the human Steroid Receptor Activator (SRA) (Lanz et al., 1999; Chooniedass-Kothari et al., 2004). SRA was initially characterized as ncRNA which co-activates steroid hormone receptors (Lanz et al., 1999) and later was revealed to also encode a functional protein (SRAP), which seems to modulate *SRA* activity (Chooniedass-Kothari et al., 2004).

**Figure 1:**
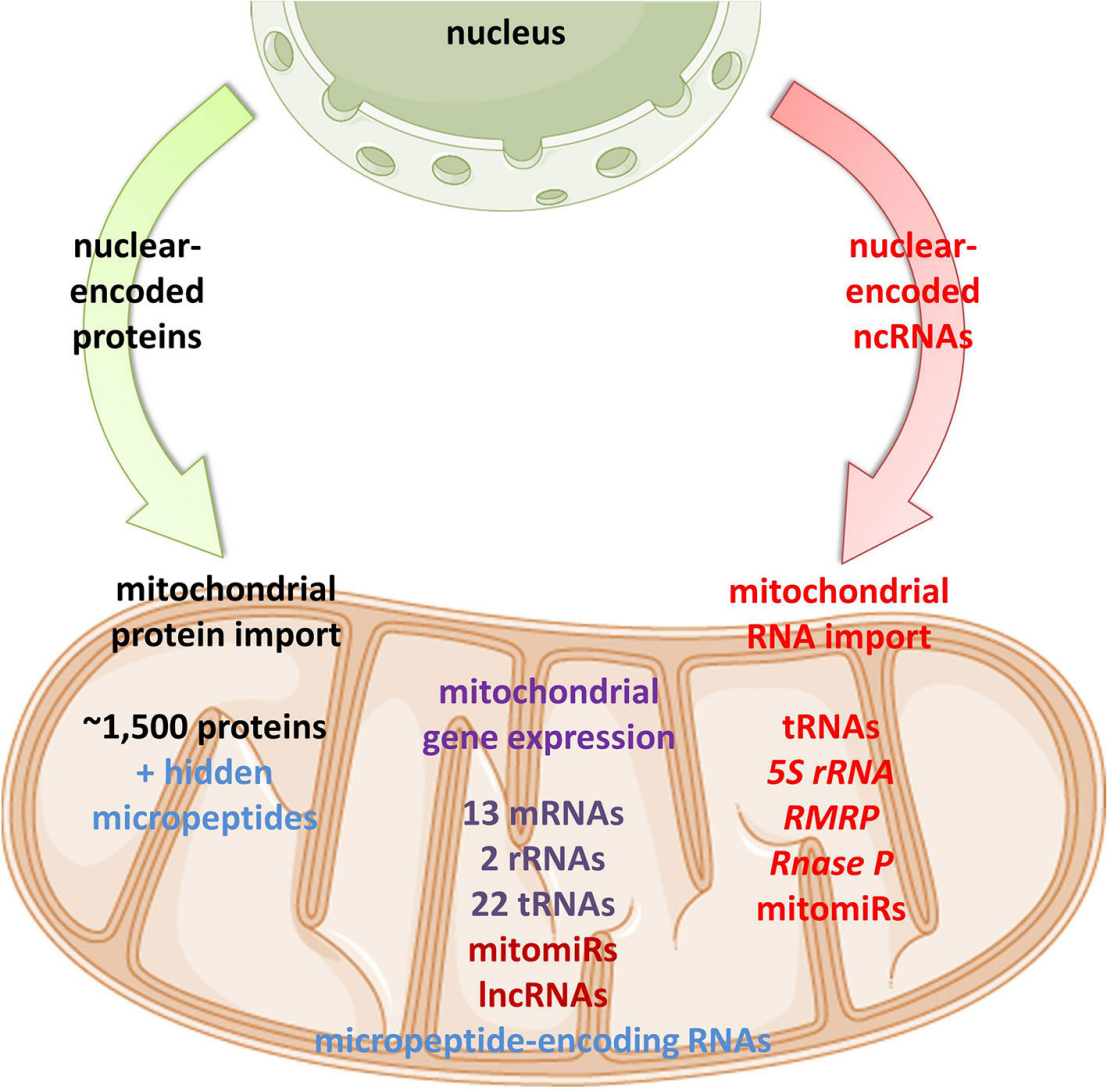
Proposed mitochondrial proteome and transcriptome. Mitochondrial homeostasis is depending on its own gene expression, but also on the import of nuclear-encoded proteins from the cytoplasm. In recent years, emerging evidence suggests import, but also mtDNA-transcription of different classes of ncRNAs in mitochondria.

**Figure 2:**
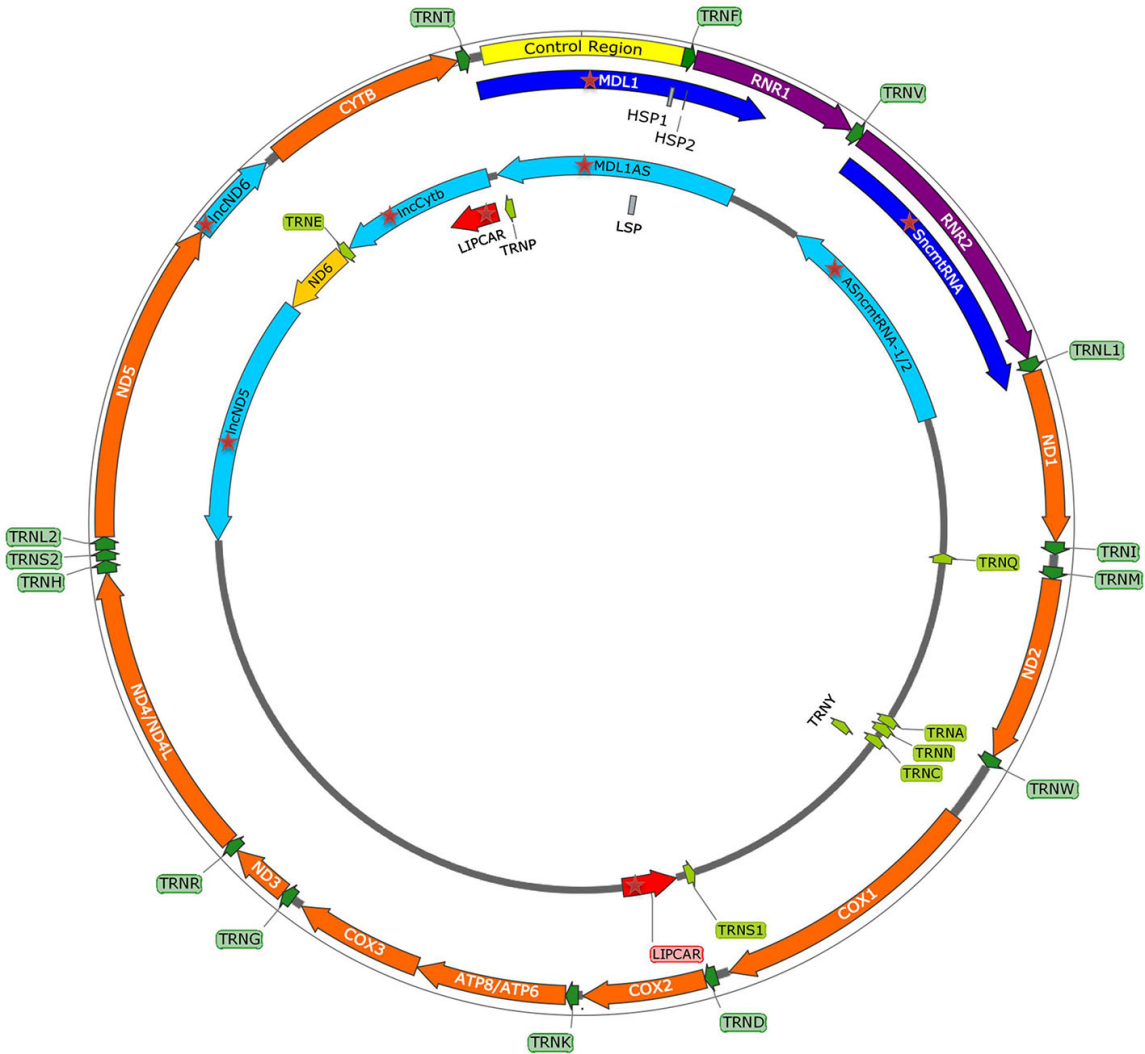
mtDNA map showing heavy (outside circle) and light (inside circle) strand and within them the control region with promoters (HSP1, HSP2, LSP), and genes encoding for 13 mitochondrial proteins, 2 rRNAs, 22 tRNAs, and recently discovered mitochondria-encoded lncRNAs (mtlncRNAs) (highlighted with red star).

Emerging discoveries in the ncRNA field have also raised the possibility that some ncRNAs affect mitochondrial biology. Mitochondria are crucial organelles for the integration of several key metabolic processes and the primary powerhouses in the cell (Spinelli and Haigis, 2018). The control of mitochondrial protein homeostasis is unique in that its components have origins in both mitochondria and nucleus (**Figure 1**). Mitochondria contain their own circular genome (mtDNA). In humans, it is 16,569 bp in length and contains 37 genes- encoding for 2 rRNAs, 22 tRNAs, and 13 proteins of the oxidative phosphorylation (OXPHOS) system (Anderson et al., 1981) (**Figure 2**). The rRNA coding sequences and all but one protein-coding sequences are separated by tRNAs and deprived of introns. The mtDNA is transcribed entirely from both strands, named heavy (H) or light (L). Transcription is initiated from the two H-strand (HSP1/2) and one L-strand promoter, located in the major non-coding region named “control region”, resulting in long polycistronic transcripts. LSP controls the transcription of eight tRNAs and the ND6 gene. HSP1 transcription produces a transcript containing tRNA^Phe^, tRNA^Val^, and the rRNAs, while transcription from HSP2 generates a transcript that spans almost the entire genome (Montoya et al., 1983; Chang and Clayton, 1984). The main proteins controlling the process are the RNA polymerase (POLRMT), two transcription factors (TFAM and TF2BM), transcription elongation factor (TEFM), and transcription termination factor (mTERF1) (Barshad et al., 2018). The “tRNA punctuation” model (Ojala et al., 1981) proposes that individual mRNA, rRNAs, and tRNAs are released from the polycistronic transcripts by the cleavage of tRNAs, which is in humans performed by endonucleases RNase P complex and ELAC2 (Holzmann et al., 2008; Brzezniak et al., 2011). After release, the rRNAs undergo chemical nucleotide modifications before becoming part of mitoribosome, the tRNAs undergo chemical nucleotide modifications, CCA addition at the 3′-end, deadenylation and finally aminoacylation, and the mRNAs get 3′ end polyadenylated (D’Souza and Minczuk, 2018). The half-life of mitochondrial transcripts and the decay of RNA intermediates are mediated by a complex of polynucleotide phosphorylase (PNPase) and SUV3 (Borowski et al., 2013). Finally, the mature mRNAs, tRNAs, and the assembled mitoribosome come together in the translation apparatus, for the synthesis of 13 subunits of OXPHOS system.

As mtDNA’s coding capacity is very limited, mitochondria are heavily dependent on the import of about 1,500 nuclear-encoded proteins. Besides, there have been indications that mitochondrial homeostasis is maintained not just through proteins, but also ncRNAs (**Figure 1**). The presence of housekeeping mitochondrial nuclear-encoded ncRNAs has been postulated for decades. These ncRNAs include tRNAs (tRNA^Leu^_UAA_, tRNA^Gln^_UUG_, tRNA^Gln^_CUG,_ tRNA^Lys^_CUU_), 5S rRNA, *RMRP,* and *RNase P* (Chang and Clayton, 1987a; Chang and Clayton, 1987b; Kiss et al., 1992; Yoshionari et al., 1994; Magalhaes et al., 1998; Puranam and Attardi, 2001; Holzmann et al., 2008). A systematic analysis of mitochondrial transcriptome further strengthened these claims. RNA-seq from 143B cells mitochondria and mitoplasts revealed the presence of several nuclear- and mitochondrial-encoded small RNAs and antisense transcripts (Mercer et al., 2011). Soon afterward, Rackham et al. (2011) observed by RNA-seq on HeLa cells that ncRNAs, excluding rRNAs and tRNAs, make up 15% of the human mitochondrial transcriptome, and identified three lncRNAs transcribed from the mtDNA. Follow-up studies have also reported the presence of ncRNAs encoded by the nuclear DNA, especially miRNAs and lncRNAs, within mitochondria across various cell types and tissues, suggesting that these ncRNAs may play important roles in the mitochondrial homeostasis (Kim et al., 2017b; Jeandard et al., 2019). The summary of the proposed nuclear-encoded ncRNAs is given in **Table 1**.

**Table 1:** Nuclear-encoded ncRNAs discovered in mitochondria.

RNA	Function in cytosol/nucleus	Proposed function in mitochondria	Evidence for mitochondrial localization	Reference
**tRNAs** (tRNA^Leu^_UAA_, tRNA^Gln^_UUG_, tRNA^Gln^_CUG,_ tRNA^Lys^_CUU_)	Translation	Translation?	RNA-seq	Rubio et al., 2008
			RT-qPCR	Mercer et al., 2011
			Enrichment in mitoplasts compared to crude mitochondria	Gowher et al., 2013
**5S rRNA**	Component of the cytosolic ribosome	Translation?	RT-qPCR and Northern blot	Yoshionari et al., 1994
			Enrichment in mitoplasts compared to crude mitochondria	Magalhaes et al., 1998
			Import into isolated mitochondria	Entelis et al., 2001
			RNA-seq	Mercer et al., 2011
			Fluorescence microscopy	Autour et al., 2018
			FISH	Zelenka et al., 2012
***RMRP***	5.8S rRNA processing	RNA metabolism?	Enrichment in mitoplasts compared to crude mitochondria	Chang and Clayton, 1987a
			RT-qPCR	Wang et al., 2010
			RNA-seq	Mercer et al., 2011
			Import into isolated mitochondria, Electron microscopy	Noh et al., 2016
***RNASE P***	Component of RNase P	Pre-tRNA processing?	RT-qPCR	Bartkiewicz et al., 1989
			Enrichment in mitoplasts in comparison to crude mitochondria	Puranam and Attardi, 2001
			Import into isolated mitochondria	Wang et al., 2010
			RNA-seq	Mercer et al., 2011
***hTERC***	Component of telomerase	Processed and transported to cytosol?	RT-qPCR	Cheng et al., 2018
**miRNAs and pre-miRNAs**	mRNA degradation/repression of mRNA translation	Repression or activation of translation, repression of transcription	RNA-seq miRNA-microarray Northern blot Enrichment in mitoplasts in comparison to crude mitochondria FISH Immunostaining	Summarized in **Table 3**
***SAMMSON***	Facilitates p32 targeting to the mitochondria in melanoma cells	?	RT-qPCR	Leucci et al., 2016
			FISH	Vendramin et al., 2018
***SRA***	Co-activates steroid hormone receptors	?	Computational screen	Baughman et al., 2009
***MALAT1***	Transcriptional regulator	Mitochondrial metabolism?	FISH	Zhao et al., 2019

Although detection of ncRNAs in mitochondria paved the way to more extensive research in this field with several examples of ncRNAs functionally described as directly impacting mitochondrial biology, these transcripts are far from being well characterized. It is important to mention that there are (still) many controversies and debates ongoing about the sole existence of ncRNA in mitochondria. The main obstacle presents the technical challenge of truly separating isolated and uncontaminated mitochondria from other membrane vesicles (endoplasmic reticulum (ER), the Golgi apparatus, the endosomes) they are tightly associated within the cell (Vendramin et al., 2017). Therefore, to assess the purity of mitochondria or mitoplasts, ER or other membrane vesicles should be used instead of cytosol or nucleus, which was not always the case. Mitoplasts—rather than mitochondria—should be subjected to RNase treatment before lysis in order to minimize the risk of contamination. Unfortunately, these control steps have not always been performed systematically, so the published data is to date a complicated topic of many debates (Vendramin et al., 2017). Moreover, implementation of high sensitive NGS techniques such as deep sequencing is likely to detect small amounts of contaminants, leading to data misinterpretation. Finally, as this field is still very fresh, many studies miss independent replicates and functional studies are published by one research group.

Despite these controversies, an increasing body of evidence has connected ncRNAs and their machinery with mitochondrial biology. In this review, we focus on classes of ncRNAs described to be functionally related with and/or localized in mitochondria: the housekeeping ncRNAs, miRNAs, and lncRNAs. We also take up the topic of mitochondrial micropeptides, recently discovered to be encoded within regions initially annotated as non-coding. Overall, we summarize knowledge on ncRNAs in mitochondrial biology and discuss their discovery, biosynthesis, import, and function in the context of both health and disease. Finally, we touch their potential as prognosis markers and therapeutic targets.

## Housekeeping ncRNAs Localized in Mitochondria

**Figure 3:**
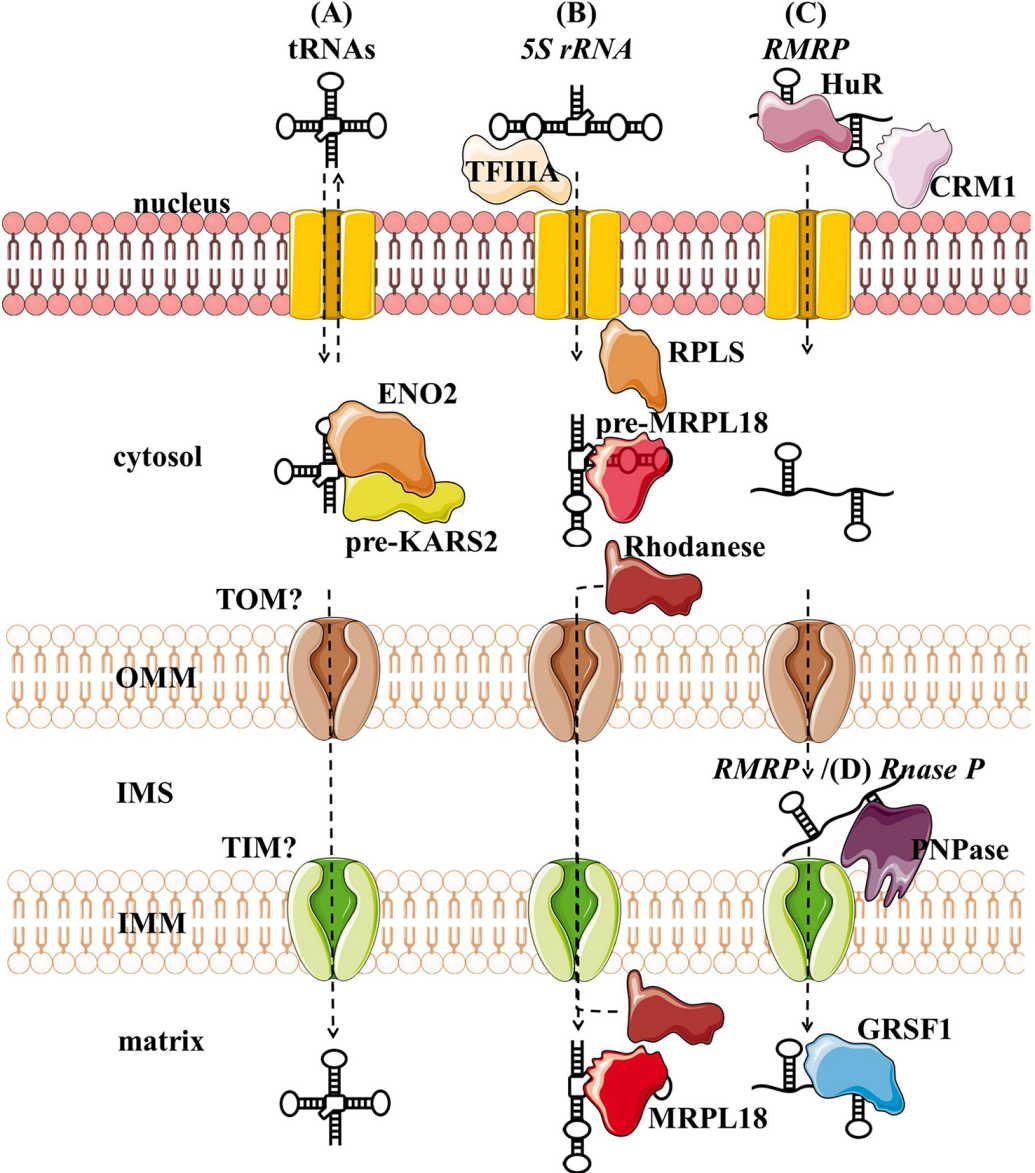
Proposed import mechanisms of tRNAs **(A)**, 5s rRNA **(B)**, and *RMRP* **(C)** into human mitochondria. ncRNAs could be targeted by various nuclear-encoded proteins localized in the nucleus and close or inside the organelle. The mechanism behind translocation across mitochondrial membranes is still unknown, but *RMRP* and *Rnase P* seem to require the PNPase **(D)**.OMM, outer mitochondrial membrane; IMS, intermembrane space; IMM, inner mitochondrial membrane.

Several tRNAs, *5S rRNA*, *RMRP,* and *RNase P* present housekeeping ncRNAs whose mitochondrial localization, transport, and function have been discussed for years. For some of them, their interacting RNA-binding proteins (RBPs) have been proposed and associated with mitochondrial import and function (**Figure 3**, **Table 1**). However, the exact import mechanism across mitochondrial membranes and the function of these ncRNAs remain unclear. It is important to note that reports of these ncRNAs have been sparse and therefore questionable, so more evidence is needed to confirm/deny their presence and role in mitochondria.

Nuclear-encoded *tRNAs* have been observed in mitochondria across many species, as most eukaryotes lack some of the essential tRNAs in their mtDNA and must import them (Tarassov et al., 2007; Schneider, 2011). Even though human mtDNA encodes all the necessary tRNAs, published data indicate that they are able to import some of the cytosolic tRNAs through conserved protein machinery. *In vitro* experiments have shown that the synthetic transcripts of yeast tRNAs could be internalized by the isolated human mitochondria (Kolesnikova et al., 2000; Entelis et al., 2001). Later, nuclear-encoded tRNAs have been detected in mitochondria (Rubio et al., 2008; Mercer et al., 2011), namely tRNA^Leu^_UAA_, tRNA^Gln^_UUG_, and tRNA^Gln^_CUG_. Gowher et al. (2013) successfully targeted yeast tRNA^Lys^_CUU_ into human mitochondria *in vivo*, suggesting similarities in the tRNA import between the two species (**Figure 3A**). The current proposal by Gowher et al. (2013) is that tRNAs are recruited from the cytosol to the mitochondria with the precursor pre-KARS2 (mitochondrial lysyl-tRNA synthetase), helped by ENO2 (glyolitic enlolase). It is still unclear how the tRNA-pre-KARS2 complex then gets internalized into the mitochondrial matrix (Gowher et al., 2013; Kim et al., 2017b). Possible protein import pathway could consist of the translocase of the outer (TOM) and inner (TIM) mitochondrial membrane, as in yeast (Tarassov and Martin, 1996). Although the import of tRNA is yet to be fully understood, it could present a novel concept for therapy for disorders caused by defects in mtDNA-encoded tRNAs. Successful import of tRNA compensating the mutated mtDNA could rescue defects in mitochondrial translation. Rescue of mtDNA mutations by the import of designed tRNAs to mitochondria has been reported *in vitro* and *in vivo* (Salinas et al., 2008; Wang et al., 2012a), but more recent reports are missing.

Several studies have suggested that *5S rRNA* is imported to the mammalian mitochondria (Yoshionari et al., 1994; Magalhaes et al., 1998). Entelis et al. (2001) suggested that mitochondrial 5S rRNA might substitute for its lost counterpart and be part of mitoribosome large subunit. Smirnov et al. (2008) proposed a model of mitochondrial 5S rRNA import (**Figure 3B**), starting with the recognition and transport of 5S rRNA from the nucleus to the cytoplasm by TFIIIA (Ciganda and Williams, 2011). In the cytosol, 5S rRNA was proposed to interact with pre-MRPL18 (precursor of mitochondrial ribosomal protein L18). This interaction might induce a conformational change in 5S rRNA that makes it recognized and bound by the mitochondrial enzyme Rhodanese, which helps it possibly translocate into mitochondria through a yet unknown mechanism. In the matrix 5S rRNA was proposed to associate with the mature MRPL18 and with mitoribosomes, affecting mitochondrial translation efficiency (Smirnov et al., 2010; Smirnov et al., 2011). However, as cryo-electron microscopy did not detect 5S rRNA within the mammalian mitoribosome 5S rRNA (Greber et al., 2015), its possible function in mitochondria remains enigmatic.

*RMRP* is a part of the RNase MRP, a ribonucleoprotein complex whose function has been discussed for decades. In the nucleus, it is involved in the pre-rRNA processing (Schmitt and Clayton, 1993; Chu et al., 1994; Goldfarb and Cech, 2017). In mitochondria, it was postulated to cleave RNA complementary to the light chain near the D-loop sites that mark the transition from RNA to DNA synthesis (Chang and Clayton, 1987b; Lee and Clayton, 1997). Three RNA-binding proteins (RBPs- HuR, PNPase, and GRSF1) have been implicated in the *RMRP* transport and role in mitochondria (**Figure 3C**). In the nucleus, *RMRP* is bound to HuR, which promotes its export to the cytosol in a CRM1-dependent manner (Noh et al., 2016). The exported *RMRP* might be then targeted into the mitochondrial intermembrane space through yet unknown mechanisms where PNPase was suggested to enable its import into the matrix (Wang et al., 2012b), after which its abundance in the matrix was reported to be increased through the interaction with GRSF1 (Noh et al., 2016). However, recent studies cast a shadow on the role of RMRP complex in mitochondria. Agaronyan et al. (2015) have shown that the RNA primer formation is a result of a premature arrest of the mitochondrial RNA polymerase after a G-quadruplex. Moreover, only the 3′ half (~130 nt) of *RMRP* could be found in mitochondria, indicating a processing that would result in a loss of catalytic activity (Esakova and Krasilnikov, 2010). These reports indicate that *RMRP* unlikely acts as an endonuclease in mitochondria. However, its interaction with GRSF1, an important component of the RNA granules (Antonicka et al., 2013; Jourdain et al., 2013), might still make it involved in the RNA metabolism.

RNase P processes the 5′ leader of precursor tRNA, which is a critical step of processing mitochondrial polycistronic transcripts (Ojala et al., 1981; Rackham et al., 2016). Two types of RNase P are known: ribonucleoproteins RNases P containing *RNase P* and protein-only RNases P (PRORP) (Lechner et al., 2015; Klemm et al., 2016). In the majority of species, including humans, it is assumed that the ribonucleoprotein RNase P acts in the nucleus and PRORP in mitochondria (Holzmann et al., 2008; Lechner et al., 2015). Strengthening this assumption, studies have reported that mammalian mitochondrial RNAse P does not require the catalytic RNA component for catalysis (Rossmanith et al., 1995; Holzmann et al., 2008). Nevertheless, *RNase P* was partially purified from HeLa cells mitochondria. Detected “*mtRNase P*”, together with the observed sensitivity of RNAse P to the nuclease treatment, suggested that RNAse P acts as a ribonucleoprotein also in mitochondria (Doersen et al., 1985). In addition, several groups indicated that *mtRNase P* is imported into the mitochondrial matrix through interaction with PNPase (Wang et al., 2010; Mercer et al., 2011; Noh et al., 2016) (**Figure 3D**). However, as so far functional RNase P ribonucleoprotein has not been reported in mitochondria, the existence of *mtRNase P* remains controversial (Jeandard et al., 2019).

*hTERC* is the RNA component of the human telomerase, where it serves as a sequence template for the telomere replication (Gall, 1990). As its sequence contains a region similar to an *RMRP* and *RNase P* short stem-loop that was proposed to enable their entry into mitochondria (Wang et al., 2010), *hTERC* was also proposed to be mitochondria-localized (Cheng et al., 2018). It was detected by the RT-PCR in purified mitoplasts, but as as a shorter, 195 nt-long transcript, which was termed TERC-53. Zheng et al. (2019) demonstrated that TERC-53 is mostly localized in the cytosol, where it regulates cellular senescence and is involved in cognition decline in mice hippocampus without affecting telomerase activity or mitochondrial functions. Having this in mind, the authors hypothesized that TERC-53 is exported from the mitochondria back to the cytosol (Cheng et al., 2018; Zheng et al., 2019). However, this hypothesis indicates *hTERC* processing occurring within the mitochondria, which has so far not been reported.

## microRNAs

**Figure 4:**
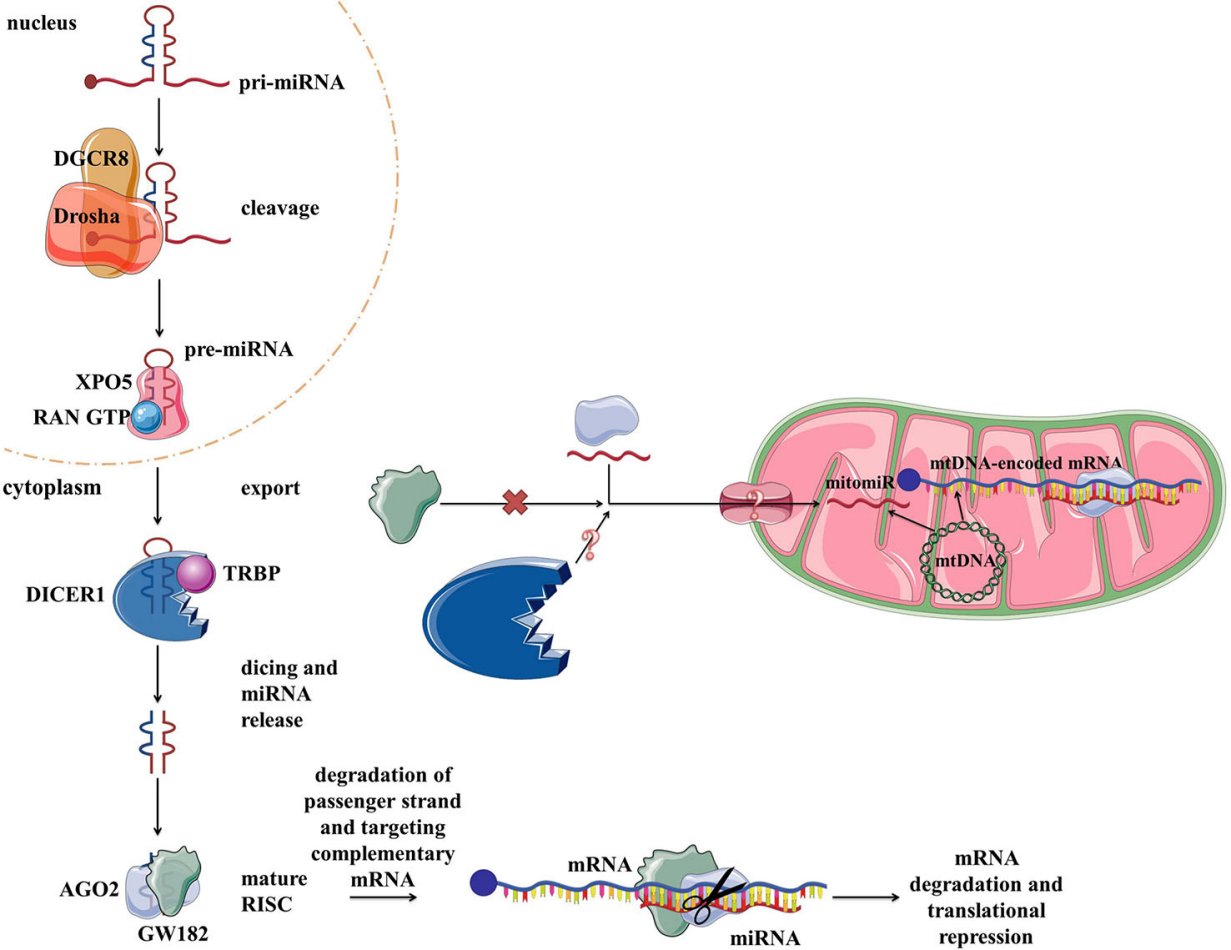
miRNA biogenesis, function in the cytoplasm within RISC, and proposed transport/presence in mitochondria. RISC, RNA-induced silencing complex.

Vertebrate genomes contain thousands of miRNAs: according to MiRBase catalog, with the human genome containing 2,654 mature sequences (Kozomara et al., 2019). The biogenesis and biological functions of miRNAs have been widely studied in eukaryotic cells (Bartel, 2009) (**Figure 4**). In short, miRNAs are transcribed from the intergenic regions or in antisense orientation to coding regions as the primary miRNA transcript (pri-miRNA). pri-miRNA is processed in the nucleus by Drosha and/or DiGeorge syndrome chromosomal region 8 (DGCR8). This results in premature miRNA (pre-miRNA) which is then bound by exportin 5 (XPO5). XPO5, along with RanGTP, enables the export of the pre-miRNA through the nuclear pore into the cytosol. There RNase Dicer (DICER1 in humans) cleaves it, producing mature double-stranded miRNA. From two strands, the “passenger strand” undergoes RNA degradation while the remaining “guide strand” associates with argonaute 2 (AGO2) and becomes part of a multiprotein RNA-induced silencing complex (RISC) (Han et al., 2006). The main function of miRNA within RISC is post-transcriptional gene regulation by promoting mRNA degradation or translational repression by sequence-specific binding to the target mRNA. mRNA degradation is achieved *via* AGO2 (Carthew and Sontheimer, 2009; Chekulaeva and Filipowicz, 2009). Translational control is mediated by GW182 (Czech and Hannon, 2011; Iwakawa and Tomari, 2015). Moreover, miRNAs have also been implicated in some non-canonical functions, such as direct transcription and chromatin state regulation in the nucleus, and even translational promotion (Vasudevan, 2012; Yao et al., 2019). Each miRNA can target multiple genes, enabling them to regulate the expression of over 60% of the human genes and therefore moderate any part of cellular biology (Bartel, 2009; Friedman et al., 2009). Focusing on mitochondria, based on their localization and genetic origin, three different classes of mitochondria-related miRNAs can be distinguished **(1)** cytoplasmic, nuclear-encoded miRNAs targeting mitochondria-related transcripts; **(2)** mitochondrial, nuclear-encoded miRNAs; and **(3)** mitochondrial, mtDNA-encoded miRNAs (Bandiera et al., 2013) (**Figure 4**). The two latter classes, termed mitomiRs, are yet to be functionally deciphered.

### Cytoplasmic miRNAs With Impact on Mitochondria

As about 1,500 nuclear-encoded proteins are imported into mitochondria and involved in diverse mitochondrial functions, many miRNAs have been described as directly targeting their mRNAs in the cytoplasm. By downregulating transcripts encoding for proteins involved in a variety of mitochondrial processes, reported miRNAs can indirectly influence mitochondrial biology and homeostasis. A summary of miRNAs reported to target nuclear-encoded mitochondrial transcripts is given in **Table 2**.

**Table 2:** miRNAs and their target genes across mitochondrial functions.

miRNA	Target	Reference
**(A) TCA cycle**		
*miR-148a*	*CS*	Tibiche and Wang, 2008
*miR-148b*	*CS*	Tibiche and Wang, 2008
*miR-299-5p*	*CS*	Tibiche and Wang, 2008
*miR-19a-3p*	*CS*	Tibiche and Wang, 2008
*miR-19b-3p*	*CS*	Tibiche and Wang, 2008
*miR-122a*	*CS*	Tibiche and Wang, 2008
*miR-421*	*CS*	Tibiche and Wang, 2008
*miR-494*	*CS*	Tibiche and Wang, 2008
*miR-183*	*IDH2*	Vohwinkel et al., 2011
*miR-743a*	*MDH2*	Shi and Gibson, 2011
*miRNA-26a*	*PDHX*	Chen et al., 2014
*miR-210*	*SDHD*	Puissegur et al., 2011
*miR-147b*	*SDHD*	Zhang et al., 2019
*miR-124*	*SUCLG2*	Wang and Wang, 2006
**(B) OXPHOS**		
*miR-101-3p*	*ATP5B*	Zheng et al., 2011
*miR-127-5p*	*ATP5B*	Willers et al., 2012
*miR-338-5p*	*ATP5G1*	Aschrafi et al., 2012
*mitomiR-378*	*ATP6*	Jagannathan et al., 2015
*miR-181c*	*COX1*	Das et al., 2014
*miR-338*	*COX4*	Aschrafi et al., 2008
*miR-34a*	*CYC*	Bukeirat et al., 2016
*miR-210-5p*	*ISCU*, *COX10*	Chan et al., 2009; Chen et al., 2010
*miR-210*	*SDHD*	Puissegur et al., 2011
*miR-147b*	*SDHD*	Zhang et al., 2019
*miR-663*	*UQCC2*	Carden et al., 2017
**(C) Fatty acid metabolism**		
*miR-204-5p*	*ACACB*	Civelek et al., 2013
*miR-224-5p*	*ACSL4*	Peng et al., 2013
*miR-122*	*Aldoa*	Esau et al., 2006
*miR-212*	*CACT*	Soni et al., 2014
*miR-132*	*CACT*	Soni et al., 2014
*miR-370*	*CPT1A*	Iliopoulos et al., 2010
*miR-33b*	*CPT1A*	Rottiers and Naar, 2012
miR-378, miR-378*	*CRAT*	Carrer et al., 2012
*miR-33a*	*CROT*	Gerin et al., 2010
*miR-107*	*PANK*	Wilfred et al., 2007
*miR-103*	*PANK*	Wilfred et al., 2007
*miR-29a-3p*	*PPARδ*	Kurtz et al., 2014
*miR-199a-5b*	*PPARδ*	el Azzouzi et al., 2013
**(D) Aminoacid metabolism**		
*miR-29b*	*DBT*	Mersey et al., 2005
*miR-23a-3p*	*GLS*	Gao et al., 2009
*miR-23b-3p*	*GLS*	Gao et al., 2009
*miR-193b*	*SHMT2*	Leivonen et al., 2011
**(E) Nucleotide metabolism**		
*miR-502*	*DHODH*	Zhai et al., 2013
*miR-940*	*MTHFD2*	Xu et al., 2019
*miR-149*	*MTHFR*	Wu C. et al., 2013
*miR-125*	*MTHFR*	Stone et al., 2011
*miR-22*	*MTHFR*	Stone et al., 2011
**(F) Mitochondrial transport**		
*miR-15b*	*Arl2*	Nishi et al., 2010
*miR-16*	*Arl2*	Nishi et al., 2010
*miR-195*	*Arl2*	Nishi et al., 2010
*miR-424*	*Arl2*	Nishi et al., 2010
*miR-25*	Mitochondrial calcium uniporter	Marchi et al., 2013
*miR-155*	*SLC25A19*	Kim et al., 2015
*miR-132*	*SLC25A20*	Soni et al., 2014
*miR-212*	*SLC25A20*	Soni et al., 2014
*miR-184*	*Slc25a22*	Morita et al., 2013
*miR-141*	*Slc25a3*	Baseler et al., 2012
**(G) Mitochondrial dynamics**		
*miR-30a-5p*	*DRP1*	Li et al., 2010
*miR-483-5p*	*Fis1*	Fan et al., 2015
*miR-484*	*Fis1*	Wang K. et al., 2012
*miR-499*	*Fnip1, Calcinurin*	van Rooij et al., 2009; Wang et al., 2011; Liu L. et al., 2016
miR-9/9*	*GTPBP3, MTO1, TRMU*	Meseguer et al., 2015
*miR-27*	*MFF*	Tak et al., 2014
*miR-761*	*MFF*	Long et al., 2013
*miR-593*	*MFF*	Fan et al., 2015
*miR-200a-3p*	*MFF*	Lee et al., 2017
*miR-140*	*MFN1*	Guan et al., 2016
*miR-19b*	*MFN1*	Li X. et al., 2014; Joshi et al., 2016
*miR-382-5p*	*MFN1, MFN2, OPA, SIRT1, PGC1-α*	Dahlmans et al., 2019
*miR-214*	*MFN2*	Bucha et al., 2015
*miR-106a*	*MFN2*	Zhang et al., 2016
*miR-195*	*MFN2*	Zhou et al., 2016
*miR-30 family*	*P53*	Li et al., 2010
*miR-149*	*PARP-2*	Mohamed et al., 2014
*miR-23a*	*PGC1-*α	Russell et al., 2013
*miR-696*	*PGC1-α*	Aoi et al., 2010
*miR-27*	*PHB*	Kang et al., 2013
*miR-494*	*TFAM*	Yamamoto et al., 2012
*miR-23b-5p*	*TFAM*	Jiang et al., 2013
*miR-590-3p*	*TFAM*	Wu et al., 2016
*miR-155-5p*	*TFAM*	Quinones-Lombrana and Blanco, 2015
*miR-200a*	*TFAM*	Yao et al., 2014
*miR-26*	*UCP1*	Karbiener et al., 2014
*miR-15a*	*UCP2*	Sun et al., 2011
*miR-133a*	*UCP2*	Chen et al., 2009
*miR-7*	*VDAC1*	Chaudhuri et al., 2016
**(H) Autophagy, mitophagy and ROS**		
*miR-146a*	*Bcl-2*	Rippo et al., 2014
*miR-181a*	*Bcl-2*	Rippo et al., 2014
*miR-195*	*Bcl-2*	Singh and Saini, 2012
*miR-24-2*	*Bcl-2*	Singh and Saini, 2012
*miR-365-2*	*Bcl-2*	Singh and Saini, 2012
*miR-497*	*Bcl-2*	Yadav et al., 2011
*miR-146*	*Bcl-2*	Zhang et al., 2017
*miR-15a*	*Bcl-2* and *Mcl-1*	Cimmino et al., 2005
*miR-16*	*Bcl-2* and *Mcl-1*	Cimmino et al., 2005
*miR-9*	*BCL2L11*	Li Y. et al., 2014
*miR-30a*	*Becn-1*	Zhu et al., 2009
*miR-17-92*	*Bim*	Molitoris et al., 2011
*miR-92a*	*Bim*	Tsuchida et al., 2011
*miR-145*	*BNIP3*	Du et al., 2017
*miR-101*	*Mcl-1*	Frankel et al., 2011
*miR-29*	*Mcl-1*	Mott et al., 2007
*miR-181*	*Mcl-1, Bcl-2*	Ouyang et al., 2012
*miR-137*	*NIX, FUNDC1*	Li W. et al., 2014
*miR-504*	*P53*	Hu et al., 2010
*miR-125b*	*P53, Bak*	Le et al., 2009; Sun et al., 2013
*miR-21*	*PTEN*	Meng et al., 2007; Zhang et al., 2010
*miR-128*	*SIRT1*	Adlakha et al., 2013
*miR-335*	*SOD2, TXNRD2*	Bai et al., 2011;
*miR-34a*	*SOD2, TXNRD2, Bcl-2, SIRT1*	Yamakuchi et al., 2008; Bai et al., 2011; Rippo et al., 2014
miR-17*	*SOD2, TXNRD2, GPX2*	Xu et al., 2010

#### TCA Cycle

**Figure 5:**
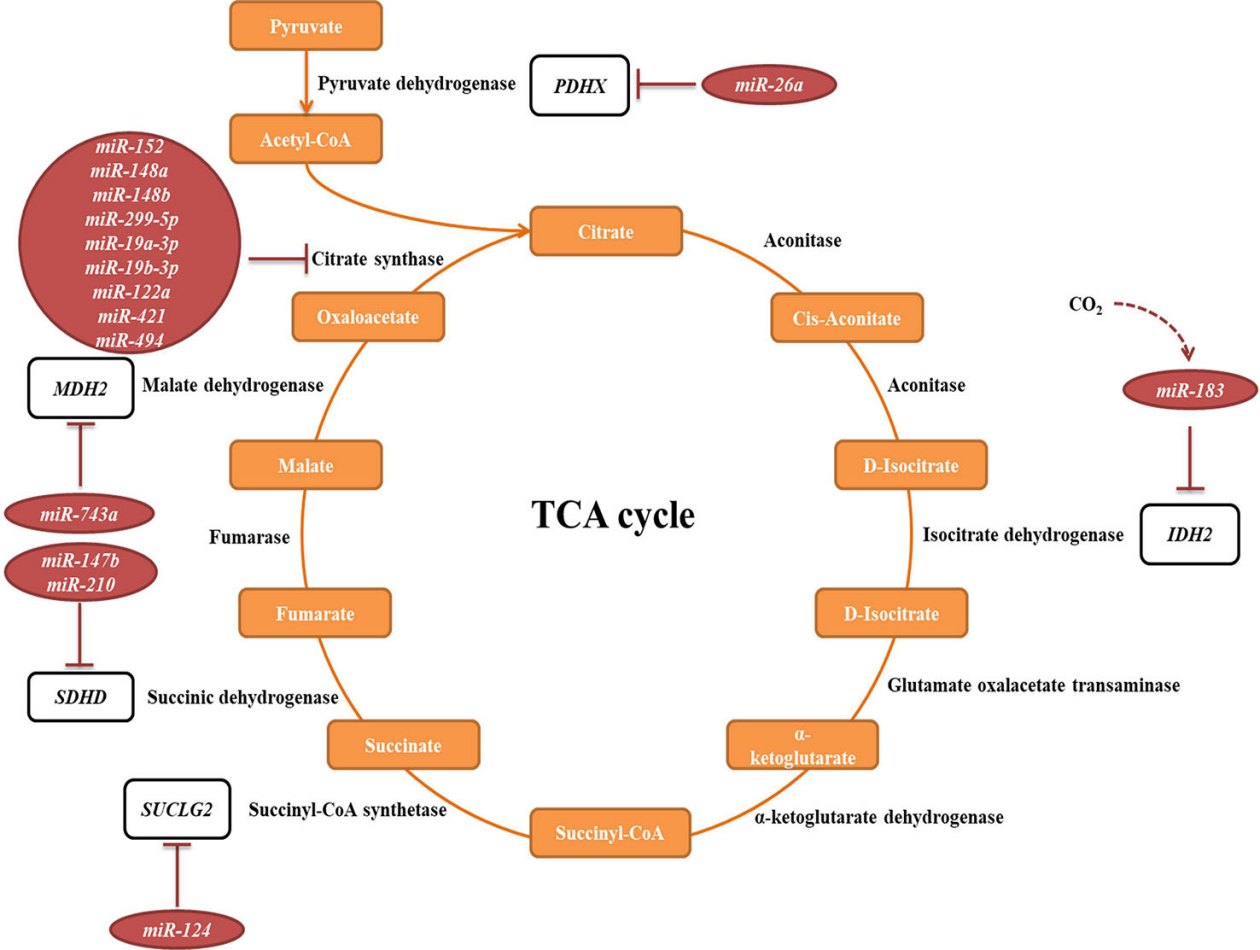
miRNAs targeting transcripts encoding proteins involved in the TCA cycle. Red arrows present the repressing effect of miRNA on its target mRNA.

The tricarboxylic acid (TCA) cycle is a central pathway in the metabolism of sugars, lipids, and amino acids. Several miRNAs have been described to directly target transcripts of enzymes involved in its chemical reactions (**Figure 5**, **Table 2A**). For example, *miR-26a* targets subunit X of pyruvate dehydrogenase (PDH). As PDH catalyzes a crucial reaction before acetyl-coA enters the TCA cycle, its repression is leading to the decreased levels of acetyl-coA and the accumulation of pyruvate (Chen et al., 2014). In cancer research, miRNAs have been discovered to have a role in developing drug tolerance. Altered *miR-147b* initiates a reversible state of tolerance to osimertinib in lung cancer cells by binding *SDHD* (Zhang et al., 2019). Pretreatment with a *miR-147b* inhibitor delayed osimertinib-associated drug tolerance, providing a promising target for preventing tumor relapse (Zhang et al., 2019).

#### Oxidative Phosphorylation System (OXPHOS)

**Figure 6:**
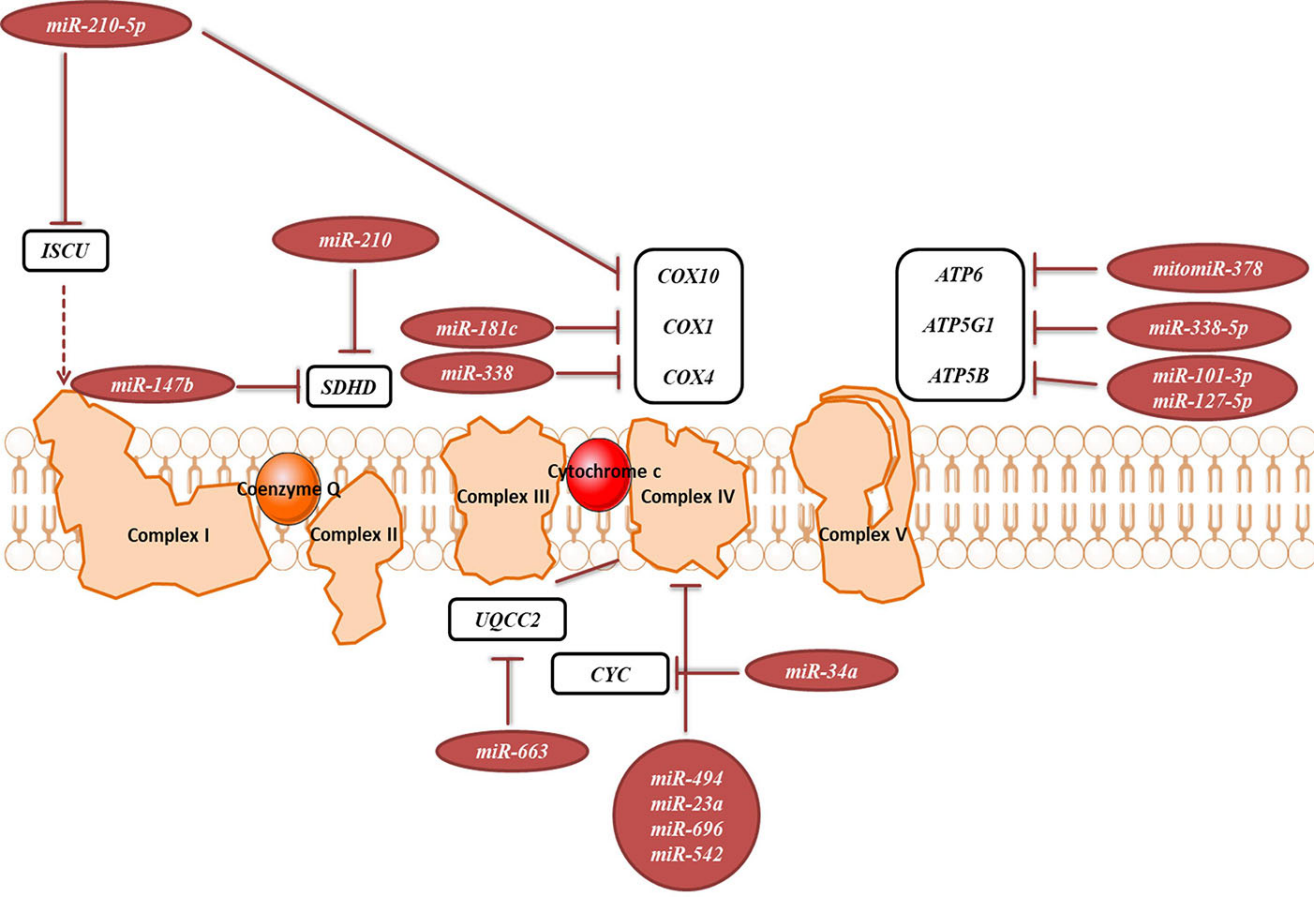
miRNAs targeting transcripts encoding proteins involved in the OXPHOS. Red arrows present the repressing effect of miRNA on its target mRNA.

OXPHOS system is composed of five protein complexes in the inner mitochondrial membrane that through oxidoreductase reactions generate a proton gradient, ultimately driving ATP synthesis. Several miRNAs have been described as directly targeting the OXPHOS subunits or assembly factors (**Figure 6**, **Table 2B**). It was shown that *miR-663* positively regulates OXPHOS subunit and assembly factor protein levels by direct stabilization of complex III assembly factor *UQCC2* (Carden et al., 2017). In breast cancer cell lines, mitochondrial dysfunction downregulates *miR-663* through hypermethylation of its promoter, which leads to decreasing OXPHOS proteins levels and enzymatic activity and stability of supercomplexes, which promotes tumorigenesis (Carden et al., 2017).

#### Fatty Acid Metabolism

Fatty acid metabolism includes catabolic and anabolic processes that involve triglycerides, phospholipids, steroid hormones, and ketone bodies. Several miRNAs have been described as regulators of these processes (**Table 2C**). As fatty acid oxidation defects have been linked to the obesity and the development of insulin resistance (Kusunoki et al., 2006), these miRNAs could serve as potential therapeutic targets. As an example, *PPARGC1B* encodes for PGC-1β, a transcriptional coactivator that promotes mitochondrial biogenesis. Interestingly, this locus can also encode for *miR-378* and miR-378*, which counterbalance the effect of PGC1-β by targeting carnitine-O-acetyltransferase (*CRAT*) (Carrer et al., 2012). miR-378/378* knockout (KO) mice showed significantly greater mitochondrial function and oxidative capacity.

#### Amino Acid Metabolism

The main steps of breakdown and synthesis of amino acids occur in mitochondria. Several miRNAs have been connected to amino acid metabolism (**Table 2D**). Most of the published work is focused on the regulation of glutaminase (GLS), which catalyzes the conversion of glutamine to glutamate. *miR-23a* and *miR-23b* participate in targeting glutaminase and thereby contribute to the mitochondrial amino acid metabolism (Gao et al., 2009).

#### Nucleotide Metabolism

Parts of the nucleotide and one-carbon metabolism are occurring in mitochondria. Various miRNAs can influence these processes (Desler et al., 2010) (**Table 2E**). For example, *miR-149*, *miR-125,* and *miR-22* have been found to target *MTHFR* (Stone et al., 2011; Wu C. et al., 2013).

#### Mitochondrial Transport

Many mitochondrial transporter and carrier proteins enable the import and export of molecules across the mitochondrial membranes. By targeting the transcripts encoding for these proteins, miRNAs are able to influence mitochondrial biology (**Table 2F**). It has been shown that the *miR-15/16* cluster, composed of *miR-15b*, *miR-16*, *miR-195*, and *miR-424*, target *Arl2* (Nishi et al., 2010).

#### Mitochondrial Dynamics

**Figure 7:**
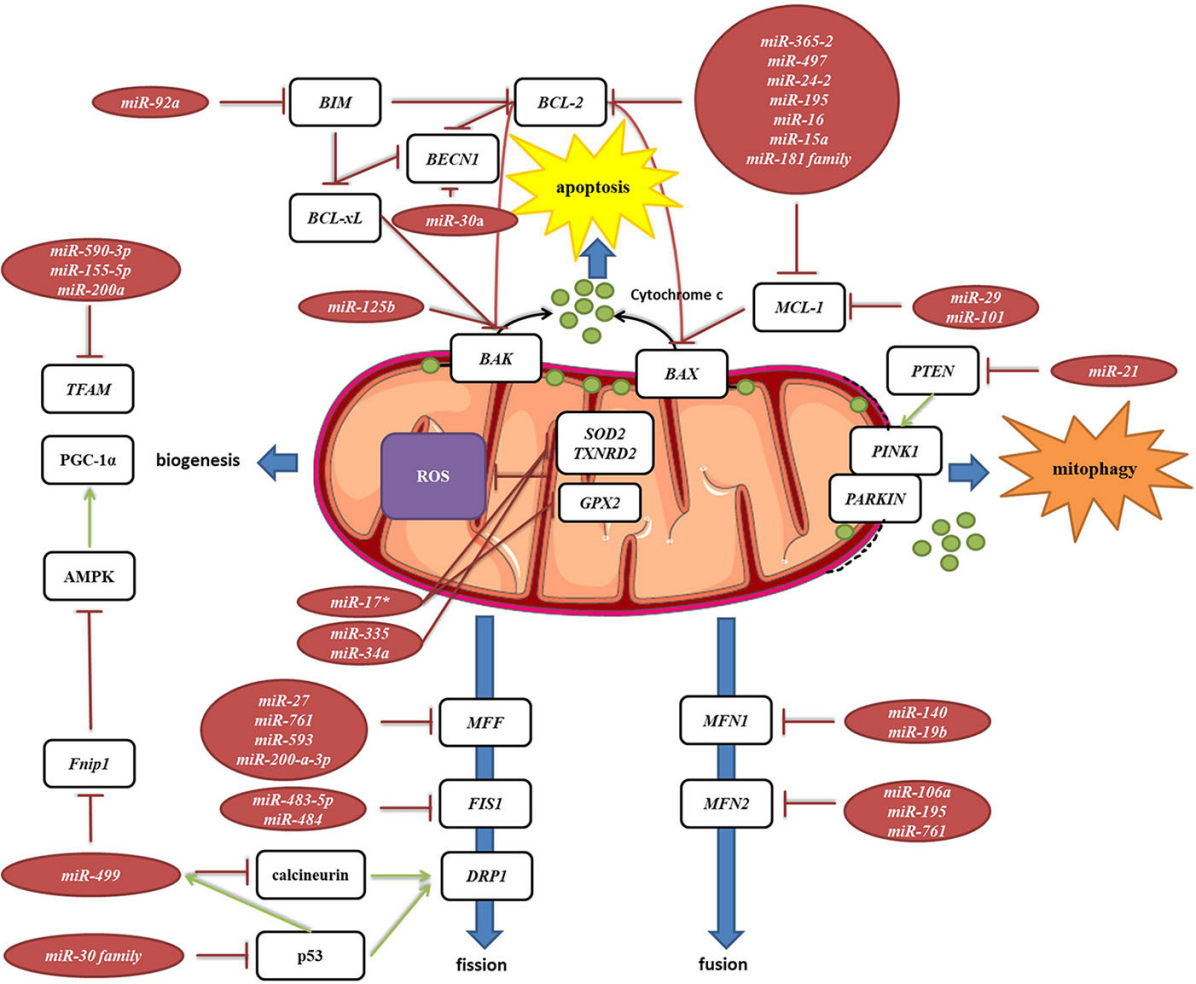
miRNA targeting transcripts encoding proteins involved in the mitochondrial dynamics, autophagy, mitophagy and ROS production. Red arrows present inhibitory effect of miRNA on its target mRNA or repressive effect of protein on its interaction partners, and green arrows present the activating effect of protein on its interaction partner.

Mitochondria are constantly changing their size, shape, and number to maximize the capacity for OXPHOS and answer the cell needs. This is achieved through the coordinated processes of biogenesis, fission, and fusion (Tilokani et al., 2018). Several miRNAs have been shown to be involved in the regulation of mitochondrial dynamics by directly or indirectly targeting these key factors (**Figure 7**, **Table 2G**). *miR-149* indirectly promotes mitochondrial biogenesis by inhibiting *PARP-2*, which increases the NAD+ levels and SIRT-1 activity, finally leading to the increased activity of PGC-1α, the master regulator of mitochondrial biogenesis. Skeletal muscles from a high fat diet-fed obese mice have low levels of *miR-149* and present with mitochondrial dysfunction, which might be due to *miR-149*-induced SIRT-1/PGC-1α pathway dysregulation. Noteworthy, miRNAs have been implicated in the mitochondria-mediated transition of skeletal muscle fiber types. *miR-499* directly targets *Fnip1*, a negative regulator of AMPK, a known activator of PGC-1 α, and thereby triggers a muscle mitochondrial oxidative metabolism program (Liu L. et al., 2016). The *miR-30* family, highly expressed in heart, was reported to regulate mitochondria fission and apoptosis by directly targeting *p53*, a transcriptional activator of Drp1 (Li et al., 2010). In addition, Drp1 is indirectly regulated by *miR-499*, which targets Drp1 activator dephosphatase calcinurin (van Rooij et al., 2009; Wang et al., 2011). Finally, *miR-499* transcription is regulated by p53 on the transcript level (Wang et al., 2011).

MELAS syndrome is caused by mutations in mtDNA affecting tRNA^Leu^_UUR_. One of the phenotypes of MELAS patients is the increased oxidative stress. In addition, mutant tRNAs^Leu^_UUR_ have reduced levels of the taurine-containing chemical modification at the wobble uridine (U34). Meseguer et al. (2015) reported that elevated oxidative stress in mutant cells leads to induction of miRNA-9/9*, which then act as post-transcriptional repressors of the tRNA-modification enzymes GTPBP3, MTO1, and TRMU. Downregulation of these enzymes disrupts the chemical modification at U34 of non-mutant tRNAs and contributes to mitochondrial dysfunction (Meseguer et al., 2015).

#### Autophagy, Mitophagy, and Reactive Oxygen Species (ROS) Production

Autophagy is a catabolic process which prevents cell damage and promotes the cell survival by degrading and/or recycling dysfunctional components during cellular stress (Dikic and Elazar, 2018). Mitophagy is a form of autophagy that removes faulty or superfluous mitochondria, regulating their number to match the cellular needs (Pickles et al., 2018). miRNAs are also involved in the mitochondria-mediated apoptosis (**Figure 7**, **Table 2H**). Moreover, they are frequently dysregulated in human cancers, where they may function as potent oncogenes or tumor suppressors (Peng and Croce, 2016). Since mitochondrial dysfunction is one of the hallmarks of cancer (Wallace, 2012), miRNAs targeting apoptosis-related transcripts could be important in the development of cancer therapies. *miR-101* (Frankel et al., 2011), *miR-30a* (Zhu et al., 2009), *miR-15a,* and *miR-16* (Cimmino et al., 2005) have been reported to target oncogenic *Bcl-2* and *Mcl-1*, and are frequently deleted or decreased in chronic lymphocytic leukemia. *miR-21* levels have been shown to be significantly increased, leading to reduced expression of PTEN in human lung and hepatocellular carcinomas (Meng et al., 2007; Zhang et al., 2010).

### mitomiRs

MitomiRs are defined as miRNAs with mitochondrial localization (Bandiera et al., 2011). The majority of mitomiRs were suggested to originate from the nuclear genome, but also there were reports of mtDNA-encoded miRNAs. Different experimental approaches across mammalian tissues and cell lines indicated the mitochondrial presence of miRNAs, but also proteins involved in miRNAs biogenesis and function, suggesting miRNAs import, transcription, and/or processing and function within mitochondria themselves. Intriguingly, mitomiRs have some unique features which distinguish them from conventional cytosolic miRNAs (Bandiera et al., 2011; Barrey et al., 2011). Most of the nuclear-encoded mitomiRs loci are located within mitochondrial gene clusters or close to mitochondrial genes, and their transcriptions are often coregulated (Baskerville and Bartel, 2005; Bandiera et al., 2011). Their size slightly differs (between 17 and 25 nt instead of the average 22 nt), and they contain short 3′ overhangs, stem-loop secondary structures, and unique thermodynamic features (Vendramin et al., 2017). They lack 5′cap and most were predicted *in silico* to target multiple mtDNA sites. It has thus been speculated that at least some of these features could present a signal for entry into mitochondria (Bandiera et al., 2011; Barrey et al., 2011).

mitomiRs have been found *via* different approaches (from miRNA microarray and RT-qPCR to deep sRNA-sequencing) and across various tissues and organisms. To begin with, sequence analysis of cDNA libraries from mice mitochondrial RNA identified clones mapping to four nuclear-encoded miRNAs and three regions within the D-loop (Lung et al., 2006). Other reports on miRNAs localized in mammalian mitochondria have expanded in the past decade (Kren et al., 2009; Bandiera et al., 2011; Barrey et al., 2011; Mercer et al., 2011; Sripada et al., 2012; Jagannathan et al., 2015), as summarized in **Table 3**. For example, Kren et al. (2009) reported by miRNA microarray 15 nuclear-encoded miRNAs from highly purified rat liver mitochondria and further strengthened their findings with Northern blot and stem-loop RT-qPCR analyses. Barrey et al. (2011) *in silico* predicted 33 pre-miRNAs and 25 miRNAs targeting mtDNA and experimentally confirmed localization of *pre-mir302a*, *let-7b,* and *mir-365* to isolated mitochondria from the human myotubes. Mercer et al. (2011) detected 31 mitochondria-encoded small RNAs in human 143B mitoplasts by sRNA-seq, the majority (84%) derived from mt-tRNA genes.

**Table 3:** miRNAs detected in mitochondria, mitomiRs.

mitomiR	Tissue	Method of detection	Reference
*Mt-1; Mt-2; Mt-3; Mt-4; let7f-;, let-7g; 122a; 101b*	Mouse liver and kidney	cDNA library	Lung et al., 2006
*130a; 130b; 140; 290; 320; 494; 671; 202; 705; 709; 721; 761; 763; 198; 765*	Rat liver	miRNA microarray, Northern blot, RT-qPCR	Kren et al., 2009
*690; 122; 451; 720; let-7f; let-7b; let-7g; 29a; 26a; 192; 101; 22; 805; 29c; 7a; 98; 26b; 30b; 7c; 709*	Mouse liver	miRNA microarray, RT-qPCR	Bian et al., 2010
*1973; 1275; 494; 513a-5p; 1246; 328; 1908; 1972; 1974;638; 1977;1978;1201*	HeLa cells	miRNA microarray, RT-qPCR	Bandiera et al., 2011
pre-mir302a; pre-let-7b; 365; 720; 133b; 1974; 24; 133a; 125a-5p; 1979; 103; 125b; 103; 221; 23a; let-7b; 423-3p; 106a; 23b; 92a; 193b; 93; 532-3p; 20a; 149; 181a; 503; 210; 107; 574-3p; 34a; let-7g; miRPlus-D1033; 19b; 197; 324-3p; 127-3p; 324-5p; 484; 151-5p; 486-5p; 542-5p; 199a-5p; 501-3p; 675*; 134; 490-3p; 598	Human myotubes	FISH, RT-qPCR	Barrey et al., 2011
*103-3p; 146a-5p; 16-5p*	143B cells	sRNA-seq	Mercer et al., 2011
*181c-5p*	Rat cardiac myocytes	miRNA microarray, immunostaining, RT-qPCR	Das et al., 2012
*107; 181a-5p; 221-5p; 320a; let-7b; let-7g*	HEK293 and HeLa cells	sRNA-seq, RT-qPCR	Sripada et al., 2012
*1*	C2C12 cells	CLIP-seq, miRACE, RT-qPCR	Zhang et al., 2014
*143-3p; 378a-3p; 146a-5p; 181c-5p; 501-3*	143B and 206 ρ° cells	sRNA-seq, RT-qPCR	Dasgupta et al., 2015
*let-7d-5p; let-7b-5p; let-7c-5p; let-7f-5p; mghv-M1-7-3p; 1187; 1224-5p; 125a-3p; 125b-5p; 126-3p; 130a-5p; 133a-3p; 133a-5p; 133b; 135a-1-3p; 139-3p; 1-3p;144-3p; 149-3p; 149-5p; 188-5p; 1894-3p; 1895; 1897-5p; 1904; 1934-3p; 1982-5p; 211-3p; 2137; 21a-5p; 22-3p; 23a-3p; 23b-3p; 24-3p; 26a-5p; 27a-3p; 27b-3p; 2861; 29a-3p; 29b-3p; 29c-3p; 3072-3p; 3081-5p; 3082-5p; 3085-3p; 3092-3p; 3095-3p; 3098-5p; 30a-5p; 30c-1-3p; 30d-5p; 30e-5p; 3102-5p; 3102-5p.2-5p; 3470a; 378a-5p; 451a; 466b-3p; 466i-5p; 483-5p; 486b; 494-3p; 497-5p; 574-5p; 652-5p; 671-5p; 680; 705; 709; 712-5p; 721; 877-3p; 99a-5p*	Mouse heart, HL-1 cells	Microarray, RT-qPCR, CLIP-seq, sRNA-seq	Jagannathan et al., 2015
*142-5p; 142-3p; 146; 150a*	Rat hippocampus, rat astrocytes	RT-qPCR	Wang et al., 2015a
*Has-mit-miR-1; Has-mit-miR-2; Has-mit-miR-3; Has-mit-miR-4; Has-mit-miR-5; Has-mit-miR-6*	Human skeletal muscle myoblasts	Northern blot, RT-qPCR	Shinde and Bhadra, 2015
*pre-miR-338*	Rat SCG neurons	qRT-PCR, co-localisation	Vargas et al., 2016
*371a-5p; 1246; 664b-3p; 513b; 4271; 2392; 4462; 1290; 4449; 3934-5p1268a*	TSCCs	miRNA microarray, RT-qPCR	Fan et al., 2019

The presence of miRNA-associated proteins in the mitochondria was only recently recognized (summarized in **Table 4**). Wang et al. (2015a) and Vargas et al. (2016) reported Dicer in the rat brain, but it was reported as absent in the mitochondria isolated from the heart (Chen et al., 2010; Das et al., 2012; Jagannathan et al., 2015). So far, only one colocalization of *pre-miR-338* and Dicer in rat brain mitochondria has been published (Vargas et al., 2016). If indeed true, the presence of Dicer could indicate that mature miRNA are formed from the precursors in mitochondria, from where they could directly affect the mitochondrial transcripts or even be exported to act in the cytosol (Bienertova-Vasku et al., 2013). However, mitochondrial localization of Dicer, Drosha, and DGCR8 has not yet been validated by other groups. Several studies have documented the presence of RNA-interference components, most notably AGO2, in the mitochondria, implying the functional importance of mitomiRs. As an example, Ago2 immunoprecipitated with miRNA from mitochondria in rat cardiac myocytes (Das et al., 2012). In addition, FXR1, a postulated RISC subunit, has been found together with Ago2 in the mitochondrial matrix of mouse cardiomyocytes (Jagannathan et al., 2015). However, an important factor for miRNA-mediated translational repression- GW182 has not been detected in any studies (Ro et al., 2013; Zhang et al., 2014). Finally, the presence of Dicer and AGO2 in mitochondria need not necessarily imply processing and function of mitomiRs, as these enzymes are involved also in other, miRNA-independent, processes (Janowski et al., 2006; Song and Rossi, 2017).

**Table 4:** miRNA biogenesis and RISC proteins detected in mitochondria.

Protein	Tissue	Method of detection	Reference
**DICER**	Rat hippocampus	Western blot, immunoprecipitation	Wang et al., 2015a
	Rat total brain, SCG neurons	Western blot, immunostaining	Vargas et al., 2016
**AGO2**	Mouse liver	Western blot	Bian et al., 2010
	HeLa cells	Western blot, immunostaining, immunoprecipitation	Bandiera et al., 2011
	Rat cardiac myocytes	Immunoprecipitation	Das et al., 2012
	HeLa cells	Immunostaining	Sripada et al., 2012
	C2C12 cells	Western blot, immunoprecipitation	Zhang et al., 2014
	143B and 206 ρ° cells	Western blot	Dasgupta et al., 2015
	Mouse cardiomyocytes, HL-1 cells	Western blot, immunoprecipitation	Jagannathan et al., 2015
	Rat hippocampus	Western blot, immunoprecipitation	Wang et al., 2015a
	TSCC	Western blot	Fan et al., 2019
**AGO3**	HEK293 cells	Immunostaining	Sripada et al., 2012
**FXR1**	Mouse cardiomyocytes	Western blot, immunoprecipitation	Jagannathan et al., 2015

**Figure 8:**
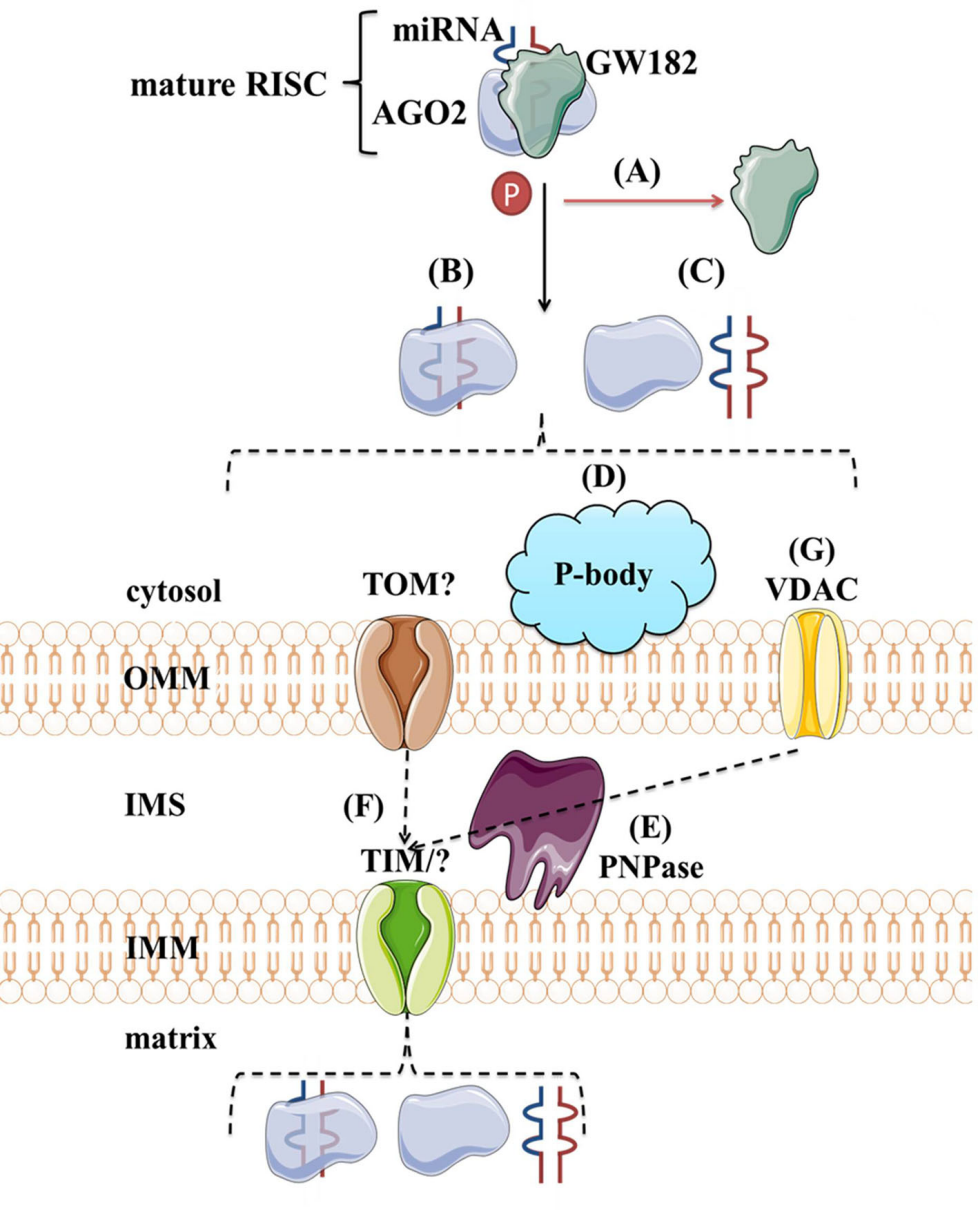
Proposed import mechanisms of miRNAs to mammalian mitochondria. A detachment of AGO2 and miRNA from RISC or just GW182 due to AGO2 phosphorylation or some other signal activation **(A)** could promote their translocation together **(B)** or separately **(C)** into the mitochondria. This process could be stimulated by P-bodies **(D)**. Translocation across mitochondrial membranes is unknown but suggested to be promoted by PNPase **(E)** and occur within TOM/TIM complexes **(F)**. Alternatively, miRNAs could rely on VDAC **(G)** at the OMM, as proposed for tRNAs in plants. OMM, outer mitochondrial membrane; IMS, intermembrane space; IMM, inner mitochondrial membrane; TOM/TIM, translocases of OMM/IMM; VDAC, voltage-gated ion channels.

Although protein transport across mitochondrial membranes is well described, the translocases for RNA transport across mitochondrial membranes remain speculative. Several mechanisms of miRNAs transport into the mitochondria have been proposed. As shown in **Figure 8**, the potential players are AGO2, processing bodies (P-bodies), polynucleotide phosphorylase (PNPase) and voltage-gated ion channels (VDAC). AGO2 has been proposed as an important factor in the subcellular localization of miRNAs. Zhang et al. (2014) have shown an association of *miR-1* with Ago2 in mitochondria and proposed their mechanism of action. At the baseline, *miR-1* is found in the cytoplasm within RISC with 3′UTR of *HDAC4*. However, during myogenesis, GW182 detaches and *HDAC4* loses 5′cap and poly(A) tail, suggesting that loss of GW182 alone or in combination with changes in *HDAC4* facilitates the transport of Ago2:*miR-1* into mitochondria (**Figure 8A**). Still, it remains unclear if AGO2 and miRNA translocate together as a complex (**Figure 8B**) or separately (**Figure 8C**) into the mitochondria and by which mechanism. Another hypothesis involves P-bodies, as they interact with mitochondria and can regulate mRNA decay, mRNA storage, and possibly miRNA import into different cellular compartments (Huang et al., 2011; Bandiera et al., 2013; Luo et al., 2018). Activation of several pathways and phosphorylation at the Ago2 Ser387 site has been shown to separate the Ago2/miRNA complex from the RISC and activate its intake into the P-body (Huang et al., 2011; McKenzie et al., 2016) (**Figure 8D**). As GW182 is also a P-body subunit (Liu et al., 2005), it might still have significance for the Ago2-miRNA import. PNPase is another candidate, as it has already been postulated to recognize specific structures of the housekeeping ncRNAs and help RNA fold properly to migrate through the mitochondrial membranes and return to its original conformation when they arrive in the mitochondrial matrix (Wang et al., 2010; Wang et al., 2012a) (**Figure 8E**). Several pre-miRNAs share the specific stem-loop structure that PNPase could recognize and enable import (Wang et al., 2010; Barrey et al., 2011; Lin et al., 2012). PNPase levels were reported to affect *mitomiR-378* mitochondrial localization and co-immunoprecipitation showed Ago2 association with PNPase, suggesting that PNPase can bind to the miRNA within the complex with Ago2 (Shepherd et al., 2017). Transport across mitochondrial membranes could occur *via* TOM/TIM complexes (**Figure 8F**). Still, additional studies are needed to prove whether and how Ago2 can go through such small pores, even if facilitated by PNPase. Finally, it has been demonstrated that VDAC, the most abundant outer mitochondrial membrane protein in plants, could help transport of tRNAs across the outer mitochondrial membrane in plant cells (Salinas et al., 2006) (**Figure 8G**). This mechanism is yet to be tested in the animal systems.

Although many have been detected, very few mitomiRs were functionally described to impact mitochondria (Baradan et al., 2017). Das et al. (2012) found *miR-181c*, Ago2, and *COX1* in mitochondrial co-immunoprecipitate, suggesting that mature *miR-181c* could translocate to mitochondria and together with Ago2 repress the translation of this mitochondrial transcript. Overexpression of *miR-181c* seems to lead to a loss of COX1 and an increase COX2 and COX3, resulting in complex IV remodeling. *miR-378* has been proposed to bind *ATP6* in mitochondria in the presence of Ago2 and FXR1, leading to a decrease of ATP6 in mouse type 1 diabetic heart (Jagannathan et al., 2015). *miR-1*, specifically induced during myogenesis, is able to promote translation of COX1 and ND1 within Ago2-miRNA complex in mitochondria, while, on the contrary, suppressing its target transcripts in the cytosol (Zhang et al., 2014). However, the binding of *miR-1* to mitochondrial transcripts has been suggested only by Ago2 CLIP experiments, and to date, *miR‐1* is the only example of this non-canonical mitomiR function. Nevertheless, as many mitochondrial diseases are caused by defects in mitochondrial translation (Pearce et al., 2013), the upregulation of mitochondrial translation *via* miRNAs may be a new therapeutic route for these diseases which currently have no cure and few treatment options. Finally, a recent report reveals the role of mitomiRs in mitochondrial transcriptional regulation. *mitomiR-2392*, together with Ago2, was reported to recognize target sequences in the H-strand and partially inhibit polycistronic mtDNA transcription in a tongue squamous cell carcinoma (TSCC) cells, leading to downregulation of oxidative phosphorylation and upregulation of glycolysis (Fan et al., 2019).

To summarize, the identification of a miRNA inside mitochondria has, without a doubt, raised the interest in studying mitomiRs. However, mitomiRs are far from being well recognized. It is initially crucial to prevent any contamination during mitochondrial/mitoplast isolation to certain their mitochondrial localization. Furthermore, the mechanisms of their import, including interaction factors and important sequence features, and functions in mitochondria are yet to be elucidated. One should be aware that mitomiRs reported across various cell types and species show a very poor overlap. This could reflect species and cell type-specific expression of mitomiRs (Geiger and Dalgaard, 2017). On the other hand, such low reproducibility raises urgent questions regarding the techniques used in the published studies (Vendramin et al., 2017). Although several hypotheses concerning miRNA import into mitochondria have been proposed, it remains without convincing experimental validation. Finally, mitomiRs mode of action in mitochondria is largely enigmatic. On the one hand, only AGO2 from RISC has been proposed to reside in the mitochondria and on the other hand, mitochondrial mRNAs contain no or very small 3′ UTRs, questioning if they can function as canonical miRNAs.

## Long Non-Coding RNAs

The number of lncRNA genes in mammals varies broadly between different sources, from less than 20,000 to more than 100,000 in humans (Zhao et al., 2016; Kopp and Mendell, 2018). According to noncode.org, they are encompassing ∼144 000 loci in humans (Zhao et al., 2016). Intriguingly, although nucleus-enriched, lncRNAs have been observed in different cell compartments, including mitochondria (Dong et al., 2017). Their biological activities are highly influenced by their localization in the cell (Mercer and Mattick, 2013; Fatica and Bozzoni, 2014). lncRNAs have been suggested to regulate cellular biology *via* transcriptional regulation, organization of nuclear domains, and bindings to proteins or other RNAs (Ulitsky and Bartel, 2013; Kopp and Mendell, 2018). It is therefore not surprising that their disruption has been associated with different diseases (Briggs et al., 2015; Huarte, 2015; Uchida and Dimmeler, 2015).

lncRNAs can be functionally classified into those that act *in cis*, and those that act *in trans* (Kopp and Mendell, 2018). ***In cis***, the lncRNA locus can regulate chromatin or gene expression of nearbye genes in at least three potential mechanisms: **(1)** DNA elements within the lncRNA promoter or locus carry the regulatory function, which is not related to the lncRNA or its production; **(2)** the act of transcription and/or splicing of the lncRNA affects nearby genes, irrespective of the transcribed lncRNA sequence; and **(3)** the lncRNA transcript alone affects the nearby genes, most commonly leading to the establishment of repressive or activating chromatin states. Some lncRNAs function *in trans* throughout the cell in, again, at least three potential mechanisms**: (1)** lncRNAs affect chromatin states and gene expression of distant genetic regions, **(2)** lncRNAs take part in the nuclear structure and organization (for example, as parts of speckles and paraspeckles), and **(3)** lncRNAs interact with proteins and/or other RNA molecules and modulate their expression and function (Lee, 2012; Rinn and Chang, 2012). Moreover, some transcripts initially annotated as lncRNAs are not non-coding, but actually encoding for biologically active *micropeptides* (Anderson et al., 2015; Matsumoto et al., 2017; Kopp and Mendell, 2018).

Over twenty lncRNAs have been described so far to affect the mitochondrial biology directly or indirectly. Some act in the cytosol, by regulating mitochondria-associated genes, often in interaction with miRNA, thus creating a complex mRNA-ncRNA regulation network. Other nuclear-encoded lncRNAs have been described to localize and act in mitochondria. As their transport mechanism into mitochondria is unknown their presence remains questionable. Finally, several lncRNAs have been discovered to be transcribed from mtDNA. These two latter mitochondria-localized, but origin-different lncRNAs could be refered to as nuclear-transported mitochondria-associated lncRNAs (ntmtlncRNAs) and mitochondria-encoded lncRNAs (mtlncRNAs) (Zhao et al., 2018).

### Cytoplasmic lncRNAs With Impact on Mitochondria

Several lncRNAs, some previously well described in the non-mitochondrial function, have been associated with mitochondrial metabolism. As in the case of miRNAs, these lncRNAs were proposed to impact a variety of mitochondrial functions by directly targeting or indirectly influencing mitochondrial-related genes/transcripts/proteins. It should be noted that most of these studies report an indirect effect of lncRNAs perturbations on mitochondria function. Besides, most of these lncRNAs were reported in the context of complex systems such as cancer. Nevertheless, they could present possible treatment strategies (De Paepe et al., 2018). A summary of these findings is given in **Table 5**, with several examples given below.

**Table 5:** Nuclear-encoded lncRNAs affecting mitochondria-related genes.

lncRNA	Target	Reference
*AK055347*	*Cyp450, ATP synthase, MSS51*	Chen G. Y. et al., 2016
*ANRIL*	*PARP, Bcl-2*	Zhu et al., 2015; Liu B. et al., 2016
*CARL*	*PHB2*	Wang et al., 2014
*BATE1*	hnRNPU	Alvarez-Dominguez et al., 2015
*CCAT2*	*GLS*	Redis et al., 2016
*Cerox*	*miR-488-3p*	Sirey et al., 2019
*ENSMUST00000136025*	*BIM*	Chen X. et al., 2016
*FAL1*	*DRP1*	Liu et al., 2019
*GAS5*	*BAX, BAK*	Gao et al., 2015
*HOTAIR*	MICU1, UQCRB	Kong et al., 2015; Zheng et al., 2015
*H19*	VDAC1	Li et al., 2016
*HOTTIP*	*GLS*	Ge et al., 2015
*MEG3*	*Bcl-2*	Wang et al., 2015b; Liu B. et al., 2016
*MPRL*	*miR-483-5p*	Tian et al., 2019
*Pvt1*	*c-Myc, Lipe, Cpt1a*	Alessio et al., 2019
*Tug1*	*PGC1-*α	Long et al., 2016
*UCA1*	*ARL2, miR-16, GLS*	Li et al., 2015; Li et al., 2017
*UIHTC*	*PGC1-*α	Zhang et al., 2018

*Cerox1* (cytoplasmic endogenous regulator of oxidative phosphorylation 1) has been described as the first direct lncRNA modulator of OXPHOS. It has been reported to positively regulate the levels of at least 12 complex I transcripts in miRNA-dependent fashion, by binding *miR-488-3p* and blocking its post-transcriptional repression of these transcripts and enabling translation. *Cerox1* knockdown was shown to decrease the enzymatic activities of complex I and IV. Accordingly, its overexpression was shown to increase their enzymatic activities and halve the cellular oxidative stress (Sirey et al., 2019).

Long et al. (2016) have described *Tug1* as a regulator of *PGC-1α* transcription in diabetic nephropathy (DN). *Tug1*-binding site was identified upstream of the Ppargc1a promoter region. *Tug1* interaction with this region recruited PGC-1α to promote its own gene transcription. *Tug1* expression was significantly repressed in the podocytes of diabetic mice and its overexpression lead to improved mitochondrial bioenergetics (Long et al., 2016).

Li et al. (2017) proposed the pro-oncogenic role of lncRNA *UCA1* in bladder tumors. *UCA1* is supposed to regulate mitochondrial function through upregulating *ARL2*, a direct target of *miR-195*. In this way, it inhibits the *miR-195* signaling pathway, leading to a tumor growth (Li et al., 2017).

### Nuclear-Transported Mitochondria-Associated lncRNAs (ntmtlncRNAs)

Several nuclear-encoded lncRNAs have been reported in mitochondria and proposed to regulate their biology (Vendramin et al., 2017; Zhao et al., 2018). However, due to a very limited number of publications and unresolved import mechanism, the presence and role of these lncRNAs are yet to be confirmed.

*SAMMSON* is predominantly expressed in aggressive melanomas, where it was described as a promoter of cell growth (Leucci et al., 2016; Vendramin et al., 2018). It has been proposed to bind to CARF and promote its binding to p32 in the cytosol (Vendramin et al., 2018). p32 is a mitochondrial and cytosolic protein that is required for the maturation of mitochondrial rRNAs (Wu H. et al., 2013), but also described as an important player in tumor metabolism (Fogal et al., 2010). Its interaction with CARF *via SAMMSON* promotes its mitochondrial targeting, where it increases protein synthesis, leading to an increased tumor cell growth (Vendramin et al., 2018). Knockdown of *SAMMSON* was shown to impair the p32 targeting to the mitochondria, resulting in mitochondrial protein synthesis defects and increased apoptosis, which could be of therapeutical potential (Leucci et al., 2016). As a fraction of *SAMMSON* was found to co-localize and co-purify with mitochondria, Leucci et al. (2016) proposed that it is accompanying p32 to the mitochondria.

The steroid receptor RNA activator (*SRA)* is an important coactivator of nuclear hormone receptors and a target for several RBPs, namely SHARP and SLIRP (Colley et al., 2008). By interaction with SRA, SHARP represses *SRA*-augmented estrogen-induced transactivation (Shi et al., 2001). SLIRP binds to the complex of *SRA* and SHARP and interferes with the repressing activity of SHARP. However, SLIRP is predominantly localized to the mitochondria (Colley et al., 2008; Pagliarini et al., 2008), where it regulates the expression, processing, and stability of mRNAs (Baughman et al., 2009; Dong et al., 2017). *SRA* and SLIRP were found in mitochondria, but their import and roles are yet to be explained (Dong et al., 2017).

Metastasis-associated lung adenocarcinoma transcript 1 (*MALAT1*) is one of the most-studied lncRNAs, mostly associated with cancer and metastasis (Wu et al., 2015; Sun and Li, 2019). Recently, Zhao et al. (2019) discovered that *MALAT1*, although normally enriched in the nucleus, to be also enriched in the mitochondria collected from HepG2 cells. *MALAT1*-deficient HepG2 cells produced less ATP and had impaired cell invasion, suggesting a role of this lncRNA in the mitochondrial metabolism (Zhao et al., 2019).

### Mitochondria-Encoded lncRNAs (mtlncRNAs)

Sets of lncRNAs have been reported to be transcribed from the mtDNA (**Figure 2**). Surprisingly, it has been noted that some of these lncRNAs seem to operate in the nucleus. However, their trafficking raises questions far beyond the current knowledge (Dietrich et al., 2015; Vendramin et al., 2017). Up to this date, the existence and functional relevance of these lncRNAs are still debatable. Mitochondria-encoded lncRNAs are divided into three categories:

1. Simple antisense mitochondrial DNA-encoded lncRNAs
  Antisense transcripts arising from the ND4 and ND6 loci were initially detected in cDNA libraries of mice mitochondria, but Northern blot failed to confirm their presence (Lung et al., 2006). Later, strand-specific RNA-seq of purified mitochondria identified *lncND5*, *lncND6*, and *lncCytb* as antisense transcripts (Mercer et al., 2011). Rackham et al. (2011) confirmed existence of these transcripts by RNA-seq and RT-qPCR, additionally revealing that they are 58%, 34% and 14% as abundant as their mRNA counterparts, respectively. These antisense RNAs create RNA-RNA duplexes with their complementary mRNAs, suggesting their role in mRNAs expression and stability (Rackham et al., 2011). Interestingly, Zhao et al. (2019) discovered that *lncCytB* is aberrantly transported to the nucleus in hepatoma HepG2 cells as compared with normal hepatic HL7702 cells, suggesting a new function of this lncRNA as a mitochondria-nuclear communicator in cancer. Furthermore, Gao et al. (2018) discovered within the PacBio full-length transcriptome dataset the lncRNA *MDL1*, which covers the tRNA^Pro^ antisense gene and the entire D-loop region, and its antisense transcript *MDL1AS*.
2. Chimeric mitochondrial DNA-encoded lncRNAs
  The first member of this class was discovered in mouse cells, comprised of the 16S rRNA linked to a 121 nucleotide 5′-leader sequence deriving from its complementary strand (Villegas et al., 2000). Afterward, similar transcript, called sense mitochondrial ncRNA (*SncmtRNA*), was identified in humans, and in this case, the mitochondrial 16S rRNA is linked to an 815 nucleotide 5′-leader sequence from its complementary strand (Villegas et al., 2007). *SncmtRNA* forms an 820 bp, double-stranded structure with a 40 nucleotide loop (Dietrich et al., 2015). Interestingly, *SncmtRNA* was only detected in the proliferating tumor but not in resting cells, suggesting that it might serve as a marker of cell proliferation (Villegas et al., 2007). Later, two antisense lncRNAs, called *ASncmtRNA-1* and *ASncmtRNA-2* were discovered. Here, the antisense mitochondrial 16S rRNA is linked to a 310 or 545 nucleotide 5′-leader sequence deriving from the complementary sense strand (Burzio et al., 2009). These two transcripts also form distinct double-stranded structures with a nucleotide loop. In contrast to *SncmtRNA*, they were detected mainly in normal cells and were much less expressed in proliferating tumor cells, suggesting their role as tumor suppressors (Burzio et al., 2009). Later, they were reported to be present in the nucleus associated with heterochromatin (Landerer et al., 2011). However, more data is needed to support this claim. It has been postulated that *ASncmtRNA-2* gets transported into the nucleus, where it presents a precursor of two miRNAs (*hsamiR-4485* and *hsa-miR-1973*), which could potentially regulate survivin, an inhibitor of apoptosis (Vidaurre et al., 2014; Bianchessi et al., 2015). Indeed, knockdown of *ASncmtRNAs* promoted apoptotic cell death due to the survivin downregulation at the translational level (Vidaurre et al., 2014).
3. Putative mitochondrial DNA-encoded lncRNAs
  These lncRNAs have been identified in the heart disease studies (Kumarswamy et al., 2014; Yang et al., 2014; Dietrich et al., 2015). RNA-seq revealed a high relative abundance (over 70%) of these transcripts in the total lncRNA population from patients with a severe heart failure (Yang et al., 2014). The most significant lncRNA has been named long intergenic noncoding RNA predicting CARdiac remodeling (*LIPCAR)*. Aligning the *LIPCAR* sequence to the human mtDNA revealed that the 5′ half aligns to the *lncCytb*, while the 3′ half aligns to the antisense region of *COX2* (Dorn, 2014). As its circulating levels were increased in the late stages of left ventricular remodeling and patients with chronic heart failure, *LIPCAR* could be used as a prognostic biomarker (Kumarswamy et al., 2014; Dietrich et al., 2015).

To conclude, lncRNAs are slowly but surely drawing attention with their complex mechanisms behind gene regulation. However, the physiological relevance of lncRNAs in mitochondria is still enigmatic. The crucial issue is the investigation of transport of the nuclear- or mtDNA-encoded lncRNAs to mitochondria and even to the nucleus. Unfortunately, there is no published data on the topic so far. Finally, the questions of specific lncRNAs mechanisms of gene regulation remain to be solved.

## lncRNA-Encoded Micropeptides

Micropeptides are a class of small peptides encoded by a sORFs, without N-terminal signaling sequence and as such are released into cytoplasm immediately after translation. Due to their sORF that escapes automatic gene annotation, they tend to be overlooked and therefore misannotated as non-coding. Indeed, lncRNAs and TUFs (transcripts of unknown function) represent the greatest source for sORFs (Yeasmin et al., 2018). Although numerous ribosome profiling studies have reported substantial ribosome occupancy of the lncRNA transcripts, the MS and the proteogenomic approaches have confirmed only a small portion of them, numbers ranging from less than 100 to up to 1600 (van Heesch et al., 2019). With a lack of consensus in the datasets, the true coding potential of lncRNAs currently remains open to speculation. Several in-depth investigations have characterized lncRNA-derived micropeptides with important roles in the ion channel modulation (Anderson et al., 2015), cell signaling (Matsumoto et al., 2017) and RNA regulation (D’Lima et al., 2017). It is important to state that the mammalian mitochondrial proteome is surprisingly enriched in micropeptides, accounting for 5% of its proteins (Calvo et al., 2016). In recent years, several micropeptides within lncRNA were discovered and characterized with a role in mitochondria, some even encoded by the mtDNA (Kim et al., 2017a). Termed mitochondrial-derived peptides (MDPs) (Kim et al., 2017a), these mtDNA-encoded peptides- humanin, MOTS-c, and SHLPs were described as potential mitochondrial bioenergetics and metabolism regulators.

*Mitoregulin* (MOXI, MPM) has been discovered by four different groups recently as a muscle- and heart-enriched 56-amino acids inner mitochondrial membrane micropeptide encoded within *LINC00116*. It has a role in mitochondrial respiratory chain supercomplexes support, fatty acids oxidation, and Ca^2+^ dynamics (Makarewich et al., 2018; Stein et al., 2018; Chugunova et al., 2019; Lin et al., 2019). Lin et al. (2019) highlighted its importance in the muscle tissue, finding it upregulated during myogenic differentiation and knockout mice exhibiting smaller skeletal muscle fibers, worse muscle performance, and slower regeneration.

*Humanin* is a 24-amino acids micropeptide whose sORF is embeded within the 16S rRNA of mtDNA (Yen et al., 2013). It was initially discovered in the surviving cells of Alzheimer’s disease brain (Hashimoto et al., 2001), suggesting its neuroprotective and cytoprotective role that has later been investigated and acknowledged across various diseases (Hashimoto et al., 2001; Muzumdar et al., 2009; Bachar et al., 2010; Oh et al., 2011; Gong et al., 2014; Kim et al., 2018). It was shown to block apoptosis, improve insulin sensitivity, decrease inflammation, and reduce oxidative stress during aging (Guo et al., 2003; Muzumdar et al., 2009; Zhao et al., 2013; Sreekumar et al., 2016). Its effects are yet to be assessed for therapeutic purposes, especially in the treatments of diabetes and neurodegenerative disorders.

*MOTS-c* (mitochondrial open reading frame of the 12S rRNA type-c) is a 16-amino acids micropeptide with an sORF within the 12S rRNA mtDNA and reported to act in the cytoplasm (Lee et al., 2015). The micropeptide was found to target the methionine-folate cycle and *de novo* purine biosynthesis pathway, increase AICAR levels, and activate AMPK, by which it increases glucose utilization, fatty acid oxidation, and changes nucleotide metabolism. MOTS-c has been proposed as a biomarker for metabolic function, as it correlates with markers of insulin resistance and obesity (Du et al., 2018). In high fat diet-induced obese mice, it prevented obesity, fat accumulation, and hyperinsulinemia, making it a possible therapeutic target (Lee et al., 2015).

*SHLPs* (small humanin-like peptides) are a group of 6 peptides discovered by an *in silico* approach to be encoded in the 16S rRNA region of mtDNA in mice (Cobb et al., 2016). Each peptide is 20-38 amino acids long, and their names were given due to similar biological effects as Humanin. Each SHLP showed a unique expression pattern across different tissues. Incubation of each synthetic SHLP with cells affected cell viability, proliferation, and apoptosis differentially, suggesting a specific role of each. Moreover, SHLP2 and SHLP3 induced oxygen consumption rate (OCR) and increased cellular ATP levels, which indicated them as mitochondrial modulators (Cobb et al., 2016). Indeed, the administration of SHLP2 to a cellular model of macular degeneration rescued its defects in the OXPHOS and the mtDNA copy number, and induced anti-apoptotic effects, indicating its therapeutic potential (Nashine et al., 2018). In addition, an intracerebral infusion of SHLP2 increased glucose uptake and suppressed hepatic glucose production (Cobb et al., 2016). Further supporting their role as insulin sensitizers, both SHLPs promoted pre-adipocyte differentiation (Cobb et al., 2016). Similarly to humanin, the circulating levels of MOTS-c and SHLP2 declined with age, indicating that they are potential regulators of aging (Lee et al., Lee et al., 2015; Cobb et al., 2016).

## Concluding Remarks

Development of high-throughput OMICS techniques, especially the next-generation sequencing, has shed new light on the non-coding fraction of the genome. Transcription of the majority of the eukaryotic genome generates not only mRNAs but a much bigger fraction of different ncRNA species that show complex structure, patterns of expression and regulation. It is now becoming apparent that RNAs are not important for cell only in the context of mRNAs as intermediates between DNA and protein, but also as powerful players themselves by affecting basically any stage of gene expression. The now expanding RNA field highlights the importance of bioinformatics analysis in order to predict and examine existence, evolution, structure, and function of non-coding regions and transcripts. Focusing on mitochondria, dozens of ncRNAs acting in the cytosol have been described to indirectly influence mitochondrial biology, usually by targeting mitochondria-related, nuclear-encoded transcripts. More surprisingly, recent research indicated that the mitochondrial transcriptome could represent a mixture of the intrinsic transcriptome and complemented by some extrinsic RNA, implying RNA import (**Figure 1**). Although dozens of papers reported ncRNAs in mitochondria, their existence is still under a question mark. Further research will need to identify their interacting partners and elucidate the molecular mechanisms behind their synthesis, transport, and function. Housekeeping ncRNAs have been proposed to have a mitochondrial localization even for decades, however, recent deeper insights into the mitochondrial biology have cast a shadow on their hypothesized role. It is clear that the re-evaluation of their presence and especially function in mitochondria is needed. Focusing on miRNA, they are well-described fine-modulators of gene regulation in the cytosol. It is not surprising that they can impact mitochondria by targeting its transcripts in the cytosol. Additionally, recent discoveries of mitomiRs suggest an attractive, even closer interplay of miRNAs and mitochondria occurring in mitochondria themselves. Yet, these findings are still a topic of many debates and therefore should be handled with caution. On the one side, the discovery of mitomiRs across different tissues and cell types by different techniques promises they are more than a false-positive finding. However, on the other side is the poor overlap between datasets that raises doubts concerning methods used. Focusing on lncRNAs, although they are among the least well-understood of these transcript species, they are slowly but surely emerging as important components of gene regulatory networks. Although the field of lncRNAs has just started to expand, published reports indicate that they influence mitochondria in different ways. Moreover, mtDNA seems to encode some lncRNAs itself. However, this field is still very fresh and further confirmation is needed, especially in the case of mitochondria-imported lncRNAs. Of clinical relevance, ncRNAs dysregulation has been noted in various mitochondria-related diseases, mostly cancer. Their association with tumorigenesis has been increasingly demonstrated. As ncRNAs often exhibit cancer-type-specific expression patterns (Iyer et al., 2015), targeting them could prove as a very selective and specific approach. Notably, they can be targeted by the antagomiRs or antisense oligonucleotides (ASOs) (reviewed by Matsui and Corey, 2017). Indeed, several pre-clinical studies have already demonstrated the therapeutic benefits of ncRNA inhibition. For example, inhibition of *SAMMSON* in melanoma xenografts suppressed the tumor growth (Leucci et al., 2016). ASOs targeting *ASncmtRNA* reduced the progression of renal adenocarcinoma and melanoma metastases in mice (Lobos-Gonzalez et al., 2016; Borgna et al., 2017). Finally, ncRNA-derived micropeptides, although biologically active as peptides, are especially interesting in terms of their discovery. As many ribosomal-profiling studies report significant ribosomal occupancy of non-coding transcripts, it is evident that further confirmation of these findings by mass spectrometry is needed in order to recognize the importance of these reported translational activities. Discoveries of mitochondrial-derived peptides and enrichment of the mammalian mitochondrial proteome in micropeptides suggest the organelle as an evolutionary playground for small proteins, either due to still unknown localization signals or import system or simply driven by the size or amino acid (positive charge) composition (van Heesch et al., 2019). This also promises that there could be many micropeptides hidden in the non-coding region, awaiting discovery and characterization. Of clinical interest, discovered mitochondria-derived micropeptides have exhibited a variety of cyto- and neuroprotective effects, and promising results of both *in vitro* and *in vivo* studies further strengthen their therapeutic potential. Overall, ncRNAs in mitochondria present a thought-provoking, but unfortunately still neglected field of study. It raises many interesting, but also challenging questions whose answers might be of clinical importance. It may reveal some enigmatic biological mechanisms (such as the RNA import in mitochondria) and eventually lead to the development of new therapeutic strategies for mitochondria-related diseases. However, before the field of ncRNA truly expands, there are still a lot of experimental approaches to be optimized and biological mechanisms to be deciphered to conclude their importance for mitochondria.

## Author Contributions

MG: conceived the topic for the review, wrote the manuscript and created the tables and figures. HP: helped shape the review, supervised the writing process, provided the critical feedback, contributed to the final version of the manuscript.

## Conflict of Interest

The authors declare that the research was conducted in the absence of any commercial or financial relationships that could be construed as a potential conflict of interest.
