# Corrigendum: ncRNAs: New Players in Mitochondrial Health and Disease?

**DOI:** 10.3389/fgene.2020.00288

**Published:** 2020-03-20

**Authors:** Mirjana Gusic, Holger Prokisch

**Affiliations:** ^1^Institute of Human Genetics, Helmholtz Zentrum München, Neuherberg, Germany; ^2^DZHK (German Centre for Cardiovascular Research), partner site Munich Heart Alliance, Munich, Germany; ^3^Institute of Human Genetics, Technical University of Munich, Munich, Germany

**Keywords:** mitochondria, ncRNA, lncRNA, miRNA, mtDNA, micropeptide

In the original article, there were mistakes in Figure 5 and Figure 6 as published. Figures are stating miR-167b instead of the correct miR-147b. The corrected Figure 5 and Figure 6 appear below.

**Figure 5:**
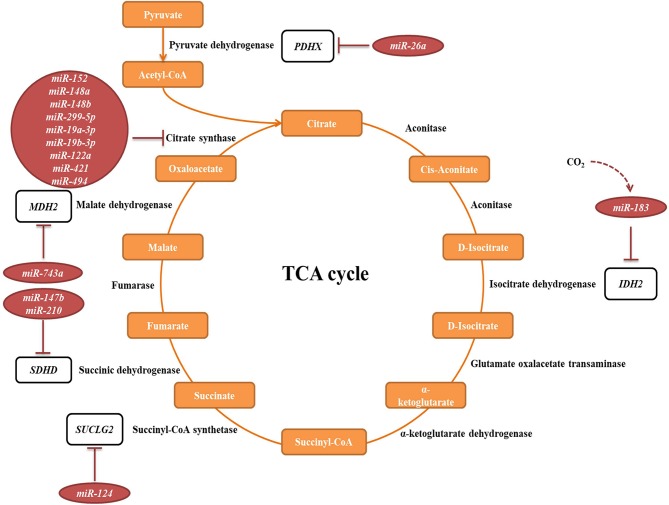
miRNAs targeting transcripts encoding proteins involved in the TCA cycle. Red arrows present the repressing effect of miRNA on its target mRNA.

**Figure 6:**
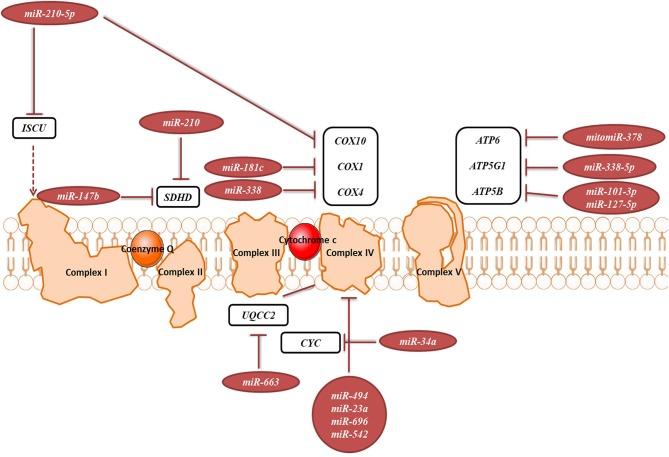
miRNAs targeting transcripts encoding proteins involved in the OXPHOS. Red arrows present the repressing effect of miRNA on its target mRNA.

The authors apologize for this error and state that this does not change the scientific conclusions of the article in any way. The original article has been updated.

